# Glucose- and glutamine-dependent bioenergetics sensitize bone mechanoresponse after unloading by modulating osteocyte calcium dynamics

**DOI:** 10.1172/JCI164508

**Published:** 2023-02-01

**Authors:** Xiyu Liu, Zedong Yan, Jing Cai, Dan Wang, Yongqing Yang, Yuanjun Ding, Xi Shao, Xiaoxia Hao, Erping Luo, X. Edward Guo, Peng Luo, Liangliang Shen, Da Jing

**Affiliations:** 1 Department of Biomedical Engineering, Fourth Military Medical University, Xi’an, China.; 2 College of Basic Medicine, Shaanxi University of Chinese Medicine, Xianyang, China.; 3 Bone Bioengineering Laboratory, Department of Biomedical Engineering, Columbia University, New York, New York, USA.; 4 Department of Neurosurgery, Xijing Hospital,; 5 State Key Laboratory of Cancer Biology, Department of Biochemistry and Molecular Biology,; 6 Ministry of Education Key Lab of Hazard Assessment and Control in Special Operational Environment, and; 7 Shaanxi Provincial Key Laboratory of Bioelectromagnetic Detection and Intelligent Perception, Fourth Military Medical University, Xi’an, China.

**Keywords:** Bone Biology, Bioenergetics, Bone disease, Calcium signaling

## Abstract

Disuse osteoporosis is a metabolic bone disease resulting from skeletal unloading (e.g., during extended bed rest, limb immobilization, and spaceflight), and the slow and insufficient bone recovery during reambulation remains an unresolved medical challenge. Here, we demonstrated that loading-induced increase in bone architecture/strength was suppressed in skeletons previously exposed to unloading. This reduction in bone mechanosensitivity was directly associated with attenuated osteocytic Ca^2+^ oscillatory dynamics. The unloading-induced compromised osteocytic Ca^2+^ response to reloading resulted from the HIF-1*α*/PDK1 axis–mediated increase in glycolysis, and a subsequent reduction in ATP synthesis. HIF-1*α* also transcriptionally induced substantial glutaminase 2 expression and thereby glutamine addiction in osteocytes. Inhibition of glycolysis by blockade of PDK1 or glutamine supplementation restored the mechanosensitivity in those skeletons with previous unloading by fueling the tricarboxylic acid cycle and rescuing subsequent Ca^2+^ oscillations in osteocytes. Thus, we provide mechanistic insight into disuse-induced deterioration of bone mechanosensitivity and a promising therapeutic approach to accelerate bone recovery after long-duration disuse.

## Introduction

Bone is a highly mechanosensitive and adaptive system, the internal structure of which can be remodeled to accommodate the prevailing mechanical environment. Load-bearing physical activity is essential for promoting and maintaining bone mass and architecture. Conversely, a lack of normal weight-bearing activity, a condition that is highly prevalent among the injured, the elderly, and astronauts, can result in significant bone loss (1, 2). The monthly rates of bone loss range from 1% to 2% during spaceflight (3), which is equivalent to the loss experienced by postmenopausal women over the course of a year (4). Individuals with long-term bed rest are also prone to a rapid loss of bone mineral density (BMD), with over 1% BMD being lost monthly in weight-bearing skeletal sites (5, 6). Notably, however, this bone loss may continue unabated even upon return to normal ambulatory conditions (reloading), and the time required for recovery is considerably greater (approximately 3–4 times longer) than the period of unloading for both astronauts and long-term bed rest patients (7–9), thereby resulting in a persistently long-duration high risk of bone fractures. Moreover, the bone quantity and quality may never fully recover to the original pre-disuse levels (7, 10, 11), suggesting that normal weight-bearing activities provide an inadequate stimulus for the restoration of bone mass. However, in contrast to the substantial volume of data relating to disuse/unloading-induced bone loss, little is currently known regarding the recovery of bone after reambulation, and as yet, no effective means of promoting such recovery has been identified. Consequently, from the perspectives of both clinical and space medicine, it is important to examine approaches that could contribute to increasing skeletal sensitivity to mechanical reloading.

Osteocytes are the most abundant (accounting for 95% of cells in the adult skeleton) and long-lived (with a lifespan of up to 50 years) of bone cells. They are deeply embedded within the mineralized bone matrix and interconnected via numerous intercellular processes to form an extensive network in the lacunocanalicular system (LCS) (12, 13). Osteocytes are previously regarded as inert space occupiers, whereas emerging evidence in recent years has tended to indicate that osteocytes may function as the major mechanosensors in bone, and may also orchestrate bone modeling and remodeling by extending their dendritic processes to communicate with osteoclasts and osteoblasts on the bone surface (14, 15). It has been established that a large number of osteocytes in both trabecular and cortical bone undergo apoptosis during spaceflight or immobilization, which contributes to the consequent activation of osteoclast-mediated bone resorption and therefore bone loss (16, 17). Given these findings indicating the important role played by osteocytes in bone mechanotransduction, it is accordingly speculated that manipulation of osteocyte viability and function might represent a potentially effective approach for attenuating unloading-induced bone loss (18). However, the mechanisms whereby osteocytes perceive and integrate the unloading and reloading cues to regulate both bone resorption and formation remain unclear.

Calcium ions (Ca^2+^) are pivotal and ubiquitous intracellular second messengers that regulate a diverse range of cellular processes, and also contribute to one of the earliest signaling events in the response of bone cells to external physical stimuli (19, 20). We have previously demonstrated that macroscopic mechanical stimuli on bone tissues induce microscopic interstitial fluid flow within the LCS, and then initiate unique intracellular Ca^2+^ oscillations in osteocytes characterized by robust repetitive Ca^2+^ spikes (21, 22). Moreover, we have established that the osteocytic networks are more dramatically responsive in Ca^2+^ dynamics than osteoblasts to mechanical stimulation (21, 22). The mechanically induced Ca^2+^ oscillations in osteocytes regulate the secretion of multiple osteocyte-related cytokines (e.g., OPG, RANKL, and sclerostin) and subsequent osteoclast and osteoblast activity (22, 23). Thus, modulating osteocyte Ca^2+^ dynamics may offer a potential opportunity to enhance the mechanosensitivity to reambulation (reloading) in bone previously subjected to long-duration disuse/unloading. Herein, we demonstrate that the capacity of the load-mediated regulation of bone microstructure, mechanical strength, and bone metabolism is compromised in skeletons previously exposed to unloading. On the basis of novel multiscale cellular Ca^2+^ imaging technologies, we found that the reduced mechanosensitivity of bone to reloading is directly associated with an attenuation of osteocytic Ca^2+^ oscillatory dynamics both in vitro and in situ. Mechanistically, the unloading-induced compromised osteocytic Ca^2+^ response to reloading was contributed from a HIF-1α/PDK1 axis–mediated specific increase in glycolysis, and a subsequent reduction in ATP production. HIF-1α was also found to transcriptionally induce substantial glutaminase 2 (GLS2) expression, and thereby exhaust the reserves of endogenous glutamine and increase the glutamine oxidation flux in osteocytes. Furthermore, inhibition of glycolysis by PDK1 blockade or glutamine supplementation restores the mechanosensitivity to reloading in skeletons previously exposed to unloading by fueling the tricarboxylic acid (TCA) cycle and rescuing subsequent Ca^2+^ oscillatory response in osteocytes.

## Results

### The mechanosensitivity of bone to reloading is significantly compromised in mice previously exposed to hind-limb unloading.

**Figure 1:**
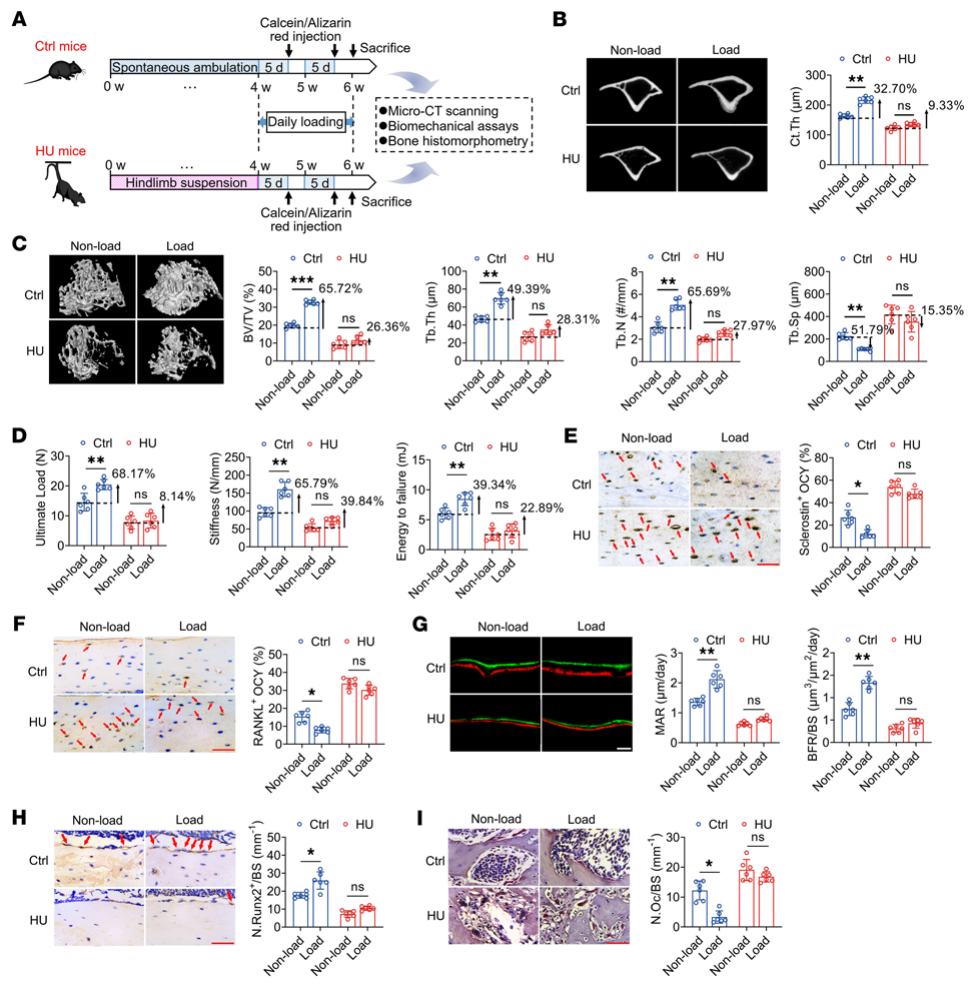
The mechanosensitivity of bone to reloading is compromised in mice previously exposed to hind-limb unloading. (**A**) Experimental protocol of 4-week tail suspension to establish the hind-limb unloading (HU) model and subsequent application of reloading with uniaxial cyclic compression (1,200 cycles/day for 2 weeks). The right tibia was subjected to daily cyclic compressive loading, and the contralateral left tibia was not mechanically loaded and was used as control (Ctrl). (**B**) Representative micro-CT images showing cortical bone thickness and the corresponding quantitative data. *n* = 6 per group. (**C**) Representative micro-CT images showing trabecular bone architecture in mouse proximal tibiae and the corresponding quantitative data. *n* = 6 per group. (**D**) Three-point bending tests for assessing whole-bone mechanical properties. *n* = 6 per group. (**E** and **F**) Immunohistochemical staining for sclerostin and RANKL expression in osteocytes (OCY) and the corresponding quantitative data. *n* = 6 per group. (**G**) Dynamic bone histomorphometry using calcein and alizarin red double labeling and the corresponding quantitative data. *n* = 6 per group. (**H**) Runx2 immunohistochemical staining labeling osteoblasts on bone surface and the corresponding quantitative data. *n* = 6 per group. (**I**) TRAP staining labeling osteoclasts on bone surface and the corresponding quantitative data. *n* = 6 per group. MAR, mineral apposition rate; BFR/BS, bone formation rate/bone surface; N. Runx2^+^/BS, number of Runx2-positive stained osteoblasts/bone surface; N. Oc/BS, osteoclast number per millimeter of trabecular bone surface. Graphs represent mean ± SD. *P < 0.05, **P < 0.01, ***P < 0.001 by 2-way ANOVA with Bonferroni’s post-test. Scale bars: **E**, **F**, **H**, and **I**, 50 μm; **G**, 30 μm.

We initially examined the effects of mechanical reloading on bone phenotype in mice that had experienced previous hind-limb unloading (HU). A HU disuse animal model was established by 4 weeks of tail suspension, followed by mechanical reloading with constrained uniaxial cyclic compression (1,200 cycles/day) on the unilateral right tibia for 2 weeks (Figure 1A and Supplemental Figure 1; supplemental material available online with this article; https://doi.org/10.1172/JCI164508DS1). Peak loads of 9.0 and 7.5 N, corresponding to 1,500 με tensile strain on the antemedial surface of the tibia, were applied in mice of the control and HU groups, respectively. As controls, we used the contralateral left tibiae, which were not subjected to mechanical reloading. Our micro-CT results revealed an approximately 50% loss of metaphyseal trabecular bone volume and a 25% loss of cortical bone in the tibia during the 4 weeks of tail suspension (Figure 1, B and C). Compared with control mice, HU mice were characterized by a less pronounced improvement in trabecular bone architecture and cortical bone thickness in response to reloading via tibial cyclic compression (i.e., the loaded tibia relative to the nonloaded side), as evidenced by a lower increase in bone volume fraction (BV/TV), trabecular number (Tb. N), trabecular thickness (Tb. Th), and cortical thickness (Ct.Th) and lower reduction in trabecular separation (Tb.Sp) (Figure 1, B and C). Furthermore, as indicated by the findings of a 3-point bending test, mechanical loading promoted a significant improvement in whole-bone mechanical properties compared with the contralateral nonloaded side in control mice (Figure 1D). However, reloading induced no significant changes in tibial mechanical strength compared with the contralateral side in the HU mice. Moreover, we also found that mechanical loading contributed to a marked suppression of osteocytic sclerostin and RANKL expression and improvement of osteocyte viability in the tibia of the control mice, but not in the HU mice (Figure 1, E and F, and Supplemental Figure 2). Similarly, mechanical loading significantly augmented the number of osteoblasts on bone surfaces and bone formation rate, and also reduced the number of osteoclasts on bone surfaces in the control mice, but not in the HU mice (Figure 1, G–I). In addition, compared with control mice, we observed that HU mice were characterized by a significant decrease in body weight and food intake from the third week of tail suspension (Supplemental Figure 2).

### The intracellular Ca^2+^ oscillatory response to reloading in osteocytes previously exposed to unloading is weakened both in vitro and in situ.

**Figure 2:**
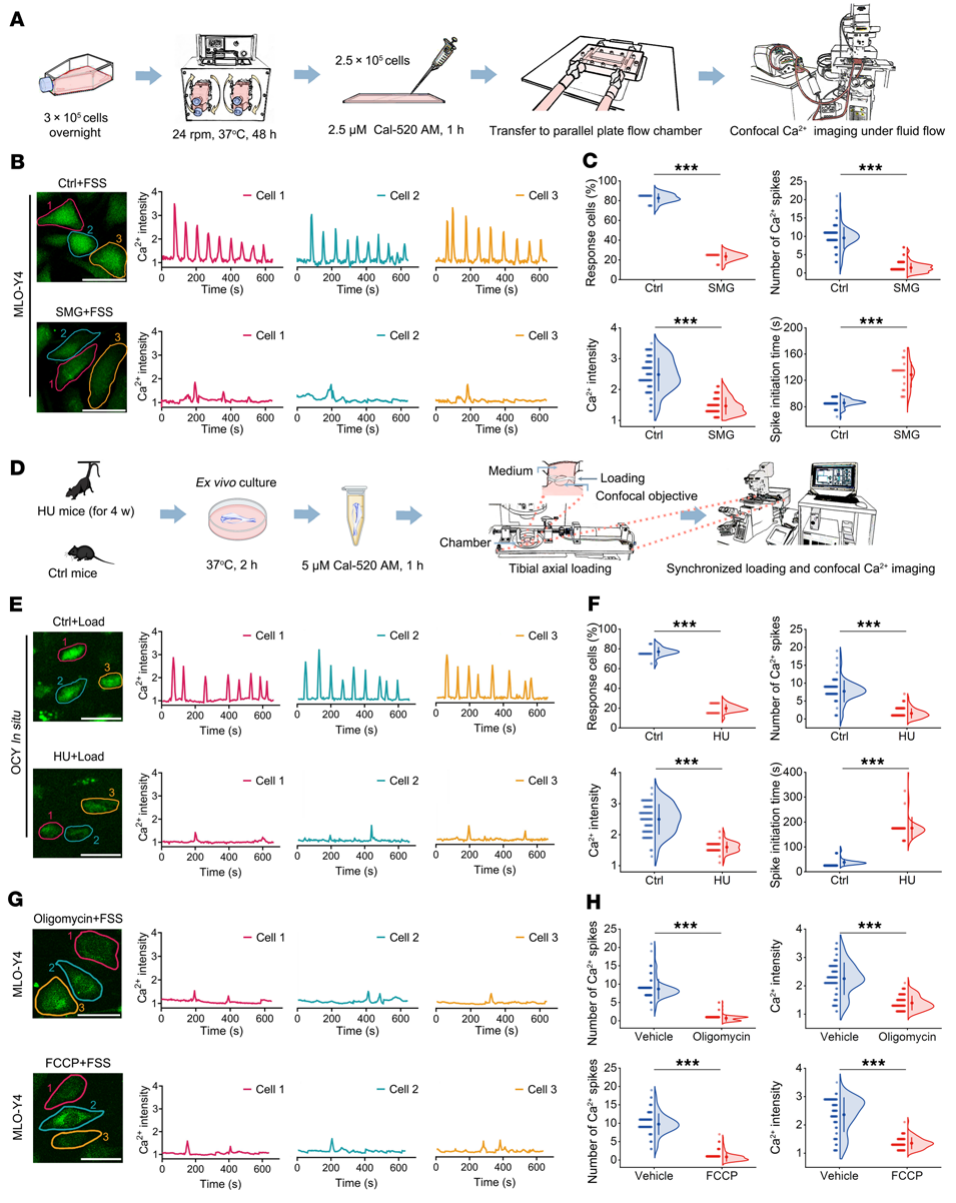
The intracellular Ca^2+^ response to reloading in osteocytes exposed to previous unloading is markedly weakened both in vitro and in situ. (**A**) The experimental protocol of osteocyte rotation to establish the in vitro model of simulated microgravity (SMG) with 24 rpm for 48 hours and subsequent real-time Ca^2+^ imaging under fluid shear stress (FSS) stimulation. (**B** and **C**) Comparison of intracellular Ca^2+^ dynamics in normal and SMG-exposed MLO-Y4 osteocytic cells in vitro in response to FSS at 2 Pa, and the corresponding quantitative data (*n* = 90 cells for Ctrl and *n* = 120 cells for SMG). (**D**) The experimental protocol of 4-week tail suspension to establish the HU model and subsequent real-time Ca^2+^ imaging in tibial osteocytes in situ under uniaxial cyclic compressive loading based on a novel synchronized cyclic loading/confocal imaging technique. (**E** and **F**) Comparison of intracellular Ca^2+^ signaling in tibial osteocytes in situ in normal and tail-suspended mice in response to subsequent uniaxial cyclic compressive loading at 1,500 με tensile strain on the antemedial surface of the tibia, and the corresponding quantitative data (*n* = 72 cells for Ctrl and *n* = 60 cells for HU). (**G** and **H**) Effects of the inhibition of mitochondrial ATP generation using oligomycin (1 μM; a mitochondrial ATP synthase inhibitor) or FCCP (1 μM; a mitochondrial oxidative phosphorylation uncoupler) on intracellular Ca^2+^ dynamics in MLO-Y4 cells in vitro under fluid flow stimulation, and the corresponding quantitative data (*n* = 85 cells for vehicle, *n* = 90 cells for oligomycin, and *n* = 70 cells for FCCP). Graphs represent mean ± SD. ***P < 0.001 by 2-tailed unpaired Student’s *t* test. Scale bars: **B** and top panel of **G**, 40 μm; **E** and bottom panel of **G**, 25 μm.

We next examined the effects of mechanical reloading on intracellular Ca^2+^ dynamics in osteocytes exposed to previous unloading both in vitro and in situ. An in vitro simulated microgravity (SMG) osteocyte model was established using a rotating bioreactor, and then real-time intracellular Ca^2+^ response to laminar fluid shear stress (FSS) was studied (Figure 2A). We accordingly observed unique Ca^2+^ oscillations with robust repetitive Ca^2+^ spikes in normal MLO-Y4 osteocytic cells in response to steady FSS stimulation (Figure 2B). Contrastingly, MLO-Y4 cells that had previously been exposed to SMG exhibited an attenuated Ca^2+^ oscillatory response with only a few weak Ca^2+^ spikes being detected (Figure 2B). Quantitative analyses showed that SMG-exposed MLO-Y4 cells had a significantly lower percentage of Ca^2+^-responsive cells, and Ca^2+^ spike number and intensity, and higher Ca^2+^ spike initiation time than normal MLO-Y4 cells in response to steady FSS stimulation (Figure 2C). We subsequently examined osteocytic Ca^2+^ dynamics in situ in intact tibia under controlled cyclic compressive loading in previous tail-suspended mice using a novel synchronized loading/confocal imaging technique (Figure 2D). We accordingly found that in contrast to the osteocytes in normal mice, which exhibited Ca^2+^ oscillations with multiple robust Ca^2+^ spikes, osteocytes in HU mice were characterized by an attenuated Ca^2+^ response to mechanical reloading (Figure 2E). The HU mice were observed to have a significantly lower percentage of responsive cells, Ca^2+^ spike number, and Ca^2+^ spike intensity, and higher spike initiation time, than the control mice (Figure 2F). Considering the importance of ATP in sustaining Ca^2+^ dynamics in many non-excitable and excitable cells (24), we examined the role of ATP in mechanically induced Ca^2+^ oscillations in osteocytes. We found that inhibition of mitochondrial ATP generation using oligomycin (a mitochondrial ATP synthase inhibitor) or carbonyl cyanide 4-(trifluoromethoxy)phenylhydrazone (FCCP; a mitochondrial oxidative phosphorylation uncoupler) almost totally abolished Ca^2+^ oscillations in MLO-Y4 cells in response to FSS stimulation (Figure 2, G and H). Then, we used pyridoxalphosphate-6-azophenyl-2′,4′-disulfonic acid (PPADS), a nonselective P_2_ receptor (P_2_R) inhibitor, to antagonize the actions of ATP at P_2_R, and thereby abolish cellular responses to ATP (25). After treatment with PPADS, osteocytic Ca^2+^ oscillatory response in MLO-Y4 cells in vitro to steady FSS stimulation and in mouse tibia in situ to cyclic compressive loading almost totally disappeared (Supplemental Figures 3 and 4). Similarly, mechanically induced osteocytic Ca^2+^ oscillations were also almost completely blocked both in vitro and in situ following inhibition of phospholipase C (the downstream molecule of P_2_R) or depletion of the ER Ca^2+^ stores using thapsigargin (Supplemental Figures 3 and 4). Our findings emphasize the importance of ATP generation and extracellular ATP-mediated Ca^2+^ release from the ER in the maintenance of osteocytic mechanical response. The ATP dependence of Ca^2+^ oscillations was confirmed in MLO-Y4 cells in response to oscillating FSS stimulation at a frequency of 1 Hz (Supplemental Figure 5).

### Unloading induces a metabolic switch from oxidative phosphorylation to glycolysis in osteocytes, resulting in a pronounced reduction in ATP synthesis.

**Figure 3:**
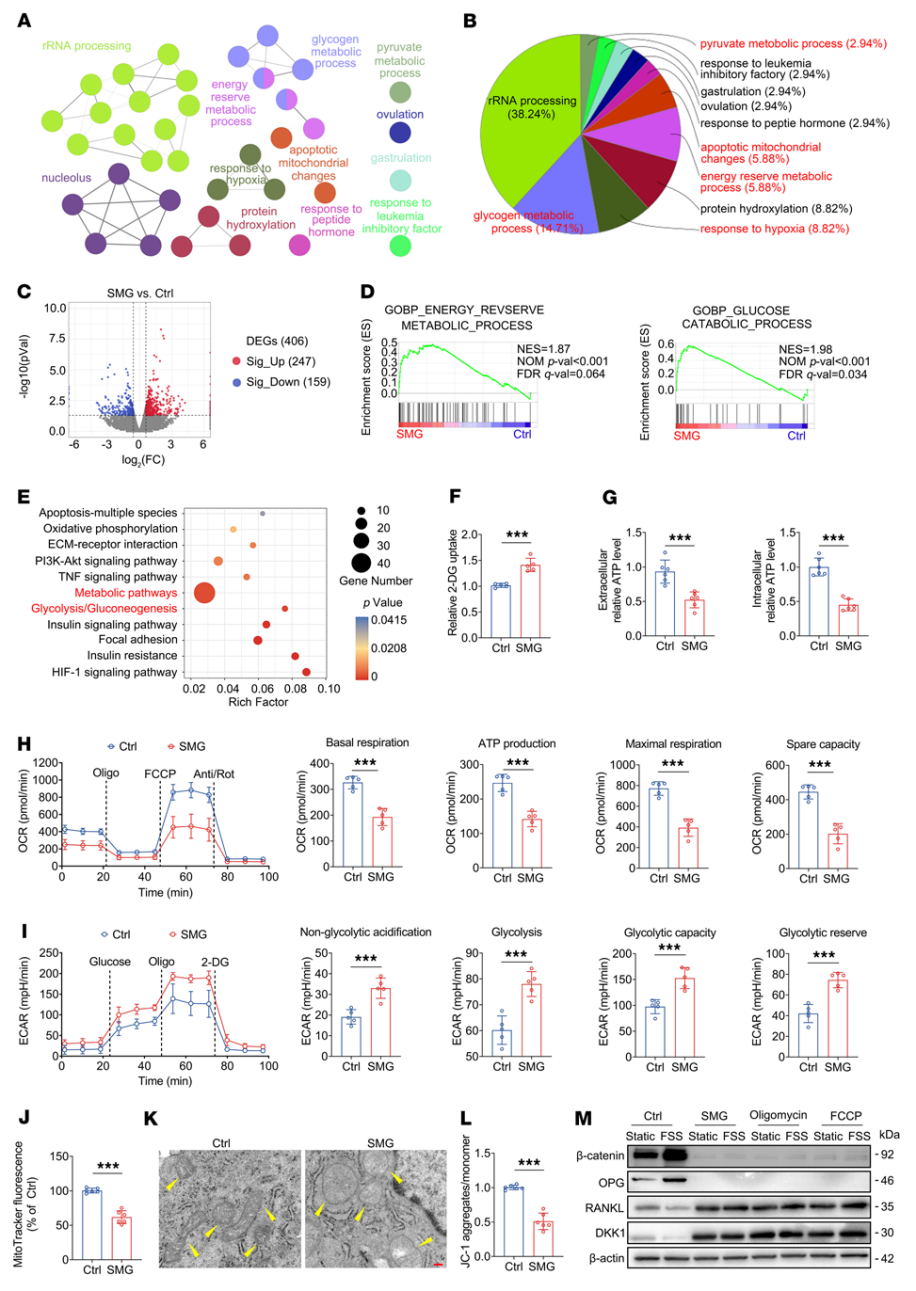
Unloading induces a metabolic switch from oxidative phosphorylation to glycolysis in osteocytes, resulting in a significant reduction in ATP synthesis. (**A**–**E**) RNA-Seq using mRNAs from MLO-Y4 osteocytic cells in vitro exposed to SMG using a rotation bioreactor with 24 rpm for 48 hours. (**A** and **B**) GO analysis showing pronounced metabolic changes in SMG-exposed MLO-Y4 cells. *n* = 3 per group. (**C**) Volcano plot showing the number of up- and downregulated genes in MLO-Y4 cells exposed to SMG compared with control cells. *n* = 3 per group. DEGs, differentially expressed genes. (**D**) GSEA showing a significant enrichment of energy metabolic process in SMG-exposed MLO-Y4 cells. *n* = 3 per group. (**E**) KEGG enrichment analysis showing significantly affected pathways in SMG-exposed cells compared with control group. Pathways with an adjusted *P* value lower than 0.05 were considered significantly enriched. *n* = 3 per group. (**F**) [^3^H]-2-DG assays for glucose uptake in normal and SMG-exposed MLO-Y4 cells. *n* = 5 per group. (**G**) Intracellular and extracellular ATP concentration assays in normal and SMG-exposed MLO-Y4 cells. *n* = 6 per group. (**H** and **I**) High-throughput Seahorse assays to simultaneously monitor cellular oxygen consumption rate (OCR) and extracellular acidification rate (ECAR) in living normal and SMG-exposed MLO-Y4 cells. *n* = 5 per group. (**J**) MitoTracker-based flow cytometry assays for assessing mitochondrial number in normal and SMG-exposed MLO-Y4 cells. *n* = 6 per group. (**K**) Transmission electron microscope assays for assessing mitochondrial shape. Yellow arrowheads indicate mitochondria. (**L**) JC-1 mitochondrial membrane potential assays in normal and SMG-exposed MLO-Y4 cells. *n* = 6 per group. (**M**) Western blotting assays of β-catenin, OPG, RANKL, and DKK1 expression in normal, SMG-exposed, oligomycin-treated (1 μM; a mitochondrial ATP synthase inhibitor), and FCCP-treated (1 μM; a mitochondrial oxidative phosphorylation uncoupler) MLO-Y4 cells in response to FSS stimulation. Graphs represent mean ± SD. ***P < 0.001 by 2-tailed unpaired Student’s *t* test. Scale bar: **K**, 200 nm.

We next elucidated the mechanisms whereby unloading induces a reduction in the mechanosensitivity of osteocytes to reloading. Gene Ontology (GO) analysis based on RNA sequencing (RNA-Seq) revealed that SMG exposure primarily induced metabolic changes in osteocytes, with glycogen metabolic process, response to hypoxia, energy reserve metabolism, and pyruvate metabolism undergoing the most pronounced changes (Figure 3, A and B, and Supplemental Figure 6). A total of 247 genes were upregulated and 159 genes were downregulated in MLO-Y4 cells after SMG exposure (Figure 3C, Supplemental Figure 6, and Supplemental Table 1). Moreover, gene set enrichment analysis (GSEA) confirmed the significant enrichment of energy metabolic process and the relevant cellular components in SMG-exposed osteocytes (Figure 3D and Supplemental Figure 6). The gene sets of bone morphogenesis, osteoclast differentiation, and osteopenia were also significantly enriched (Supplemental Figure 6). Kyoto Encyclopedia of Genes and Genomes (KEGG) pathway enrichment analysis showed that among those pathways that were enriched, metabolic pathways and glycolysis/gluconeogenesis were notably affected by SMG (Figure 3E). Compared with the normal MLO-Y4 cells, radiolabeled [1,2-^3^H]-2-deoxyglucose ([^3^H]-2-DG; a glucose analog) and 2-(*N*-(7-nitrobenz-2-oxa-1,3-diazol-4-yl)amino)-2-deoxyglucose (2-NBDG; a fluorescent glucose analog for visualizing glucose uptake in living cells) assays revealed an increase in glucose uptake in MLO-Y4 cells that had been exposed to SMG (Figure 3F and Supplemental Figure 6). Consistent with the [^3^H]-2-DG and 2-NBDG results, the mRNA and protein expression of glucose transporter 1 (GLUT1) was significantly higher in SMG-exposed MLO-Y4 cells than in the control cells (Supplemental Figure 6). Furthermore, SMG resulted in a significant reduction in the concentrations of both intracellular and extracellular ATP and a significant increase in intracellular ADP and AMP levels in MLO-Y4 cells (Figure 3G and Supplemental Figure 6). We subsequently performed high-throughput Seahorse assays to simultaneously monitor the intact cellular oxygen consumption rate (OCR) and extracellular acidification rate (ECAR) in living cells, on the basis of which we observed that, compared with control cells, SMG-exposed MLO-Y4 cells exhibited a significant decrease in OCR levels, as evidenced by lower levels of basal respiration, ATP production, maximal respiration, and spare capacity in SMG-exposed cells (Figure 3H). Furthermore, SMG resulted in a significant increase in ECAR levels in MLO-Y4 cells, including non-glycolytic acidification, glycolysis, glycolytic capacity, and glycolytic reserve (Figure 3I). Consistently, the RNA-Seq and quantitative real-time PCR (qRT-PCR) results revealed a significant increase in the expression of glycolysis-related genes after SMG exposure, such as HK1/2, Pfkp, and Ldha (Supplemental Figure 6). SMG also resulted in a significant decrease in TCA cycle metabolites in MLO-Y4 cells, including acetyl-CoA and α-ketoglutarate (α-KG) levels (Supplemental Figure 6). The MitoTracker-based flow cytometry assays (MedChemExpress, catalog HY-135056) demonstrated that SMG induced a significant decrease in mitochondrial mass in MLO-Y4 cells (Figure 3J). The transmission electron microscope revealed that SMG-exposed MLO-Y4 cells underwent mitochondrial swelling and loss and lysis of mitochondrial crest in contrast to normal cells (Figure 3K). Moreover, SMG exposure resulted in a significant decrease in mitochondrial membrane potential (Figure 3L). FSS-induced increase in β-catenin and OPG expression and decrease in RANKL and DKK1 expression were suppressed after inhibition of mitochondrial ATP generation using oligomycin or FCCP in MLO-Y4 cells, similarly to what was observed in SMG-exposed cells (Figure 3M). SMG-induced deterioration of intracellular Ca^2+^ oscillatory response to reloading and increase in glycolysis were confirmed in primary osteocytes (Supplemental Figure 7). Collectively, our findings thus provide evidence to indicate that unloading induces a metabolic shift from oxidative phosphorylation to glycolysis in osteocytes, resulting in a pronounced reduction in ATP synthesis.

### Unloading increases glycolysis in osteocytes via the specific activation of the HIF-1α/PDK1 axis.

**Figure 4:**
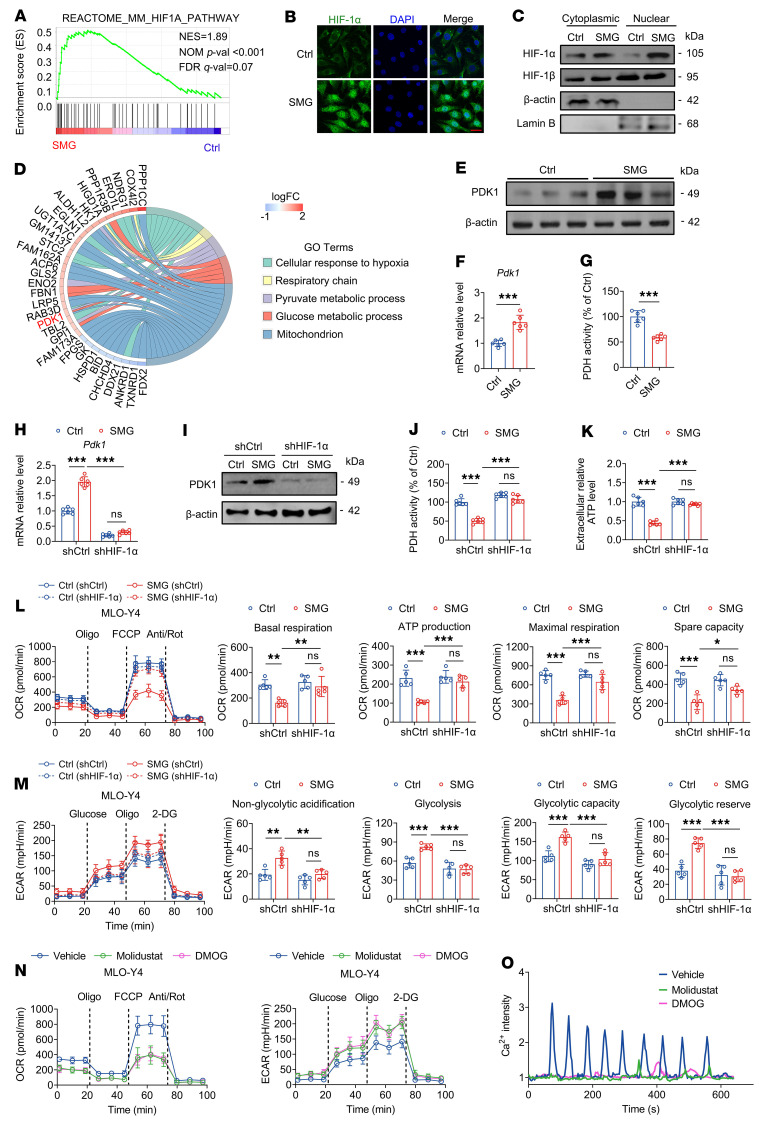
Unloading increases osteocytic glycolysis via specific activation of the HIF-1α/PDK1 axis. (**A**) GSEA based on RNA-Seq showing significant enrichment of the HIF-1α pathway in SMG-exposed MLO-Y4 osteocytic cells. *n* = 3 per group. (**B** and **C**) Immunofluorescence and Western blotting assays showing significant HIF-1α nuclear translocation in MLO-Y4 cells after SMG exposure. (**D**) GO chord graph presentation showing that PDK1 is a significantly upregulated gene in SMG-exposed MLO-Y4 cells. *n* = 3 per group. (**E** and **F**) qRT-PCR and Western blotting assays of PDK1 gene and protein expression in normal and SMG-exposed MLO-Y4 cells. *n* = 6 per group. (**G**) PDH activity assay in normal and SMG-exposed MLO-Y4 cells. *n* = 6 per group. (**H** and **I**) qRT-PCR and Western blotting assays of the PDK1 gene and protein expression in normal and SMG-exposed MLO-Y4 cells infected with shCtrl and shHIF-1α lentivirus. *n* = 6 per group. (**J** and **K**) PDH activity and extracellular ATP concentration assays in normal and SMG-exposed MLO-Y4 cells infected with shCtrl and shHIF-1α lentivirus. *n* = 6 per group. (**L** and **M**) High-throughput Seahorse assays to monitor cellular OCR and ECAR in normal and SMG-exposed MLO-Y4 cells infected with shCtrl and shHIF-1α lentivirus. *n* = 5 per group. (**N**) Effects of pharmacological activation of HIF-1α using molidustat (10 μM) or DMOG (1 mM) on cellular OCR and ECAR in normal MLO-Y4 cells. *n* = 5 per group. (**O**) Effects of molidustat or DMOG treatment on intracellular Ca^2+^ dynamics in normal MLO-Y4 cells under FSS stimulation. Graphs represent mean ± SD. (**F** and **G**) ***P < 0.001 by Student’s *t* test. (**H**–**M**) *P < 0.05, **P < 0.01, ***P < 0.001 by 2-way ANOVA with Bonferroni’s post-test. Scale bar: **B**, 25 μm.

On the basis of the aforementioned findings, we proceeded to further examine the mechanisms whereby unloading induces a substantial increase in osteocyte glycolysis. The results of both KEGG and GSEA revealed a significant enrichment of the HIF-1α pathway in SMG-exposed MLO-Y4 cells (Figure 3E and Figure 4A). Moreover, the immunofluorescence and Western blotting results revealed a higher nuclear expression of HIF-1α in SMG-exposed MLO-Y4 cells than in control cells, thereby indicating the SMG-induced nuclear translocation of HIF-1α (Figure 4, B and C). However, SMG exposure induced no significant change in HIF-1β expression in MLO-Y4 cells (Figure 4C). Furthermore, chord diagram analysis based on RNA-Seq revealed that pyruvate dehydrogenase kinase 1 (PDK1), a known key target gene of HIF-1α, was significantly upregulated in 4 GO terms related to hypoxia and energy metabolism in SMG-exposed MLO-Y4 cells (Figure 4D). PDK1 can inhibit the conversion of pyruvate to acetyl-CoA by primarily inactivating TCA cycle enzyme, e.g., pyruvate dehydrogenase (PDH), resulting in a switch of glucose metabolism from mitochondrial oxidation to aerobic glycolysis (26). qRT-PCR and Western blotting results confirmed the SMG-induced upregulation of PDK1 expression at both gene and protein levels (Figure 4, E and F). ELISA assays revealed that PDH activity was markedly reduced in SMG-exposed MLO-Y4 cells (Figure 4G). Then, we introduced HIF-1α lentivirus constructs into MLO-Y4 osteocytic cells and primary osteoblasts (the knockdown efficiency is shown in Supplemental Figure 8) to examine the effects of HIF-1α silencing on glucose metabolism. We found that the SMG-induced increase in gene and protein expression of PDK1 and decrease in PDH activity were significantly suppressed after blockade of HIF-1α (Figure 4, H–J). According to ATP measurements and Seahorse assay results, the SMG-induced reductions in ATP production and oxidative phosphorylation and an increase in glycolysis were also abolished after HIF-1α silencing in MLO-Y4 cells (Figure 4, K–M). However, we found that HIF-1α silencing induced no obvious changes in the levels of either OCR or ECAR in SMG-exposed primary osteoblasts (Supplemental Figure 9). Conversely, pharmacological activation of HIF-1α in MLO-Y4 cells using molidustat and dimethyloxaloylglycine (DMOG), two competitive inhibitors of HIF-hydroxylated prolyl hydroxylase, resulted in a significant decrease in OCR levels and increase in ECAR levels in comparison with control cells (Figure 4N). Moreover, after treatment with molidustat or DMOG, intracellular Ca^2+^ oscillatory response in MLO-Y4 cells to FSS stimulation almost totally disappeared (Figure 4O), similarly to that observed in SMG-exposed MLO-Y4 cells.

### HIF-1α induces substantial GLS2 expression and glutamine addiction in osteocytes with previous unloading.

**Figure 5:**
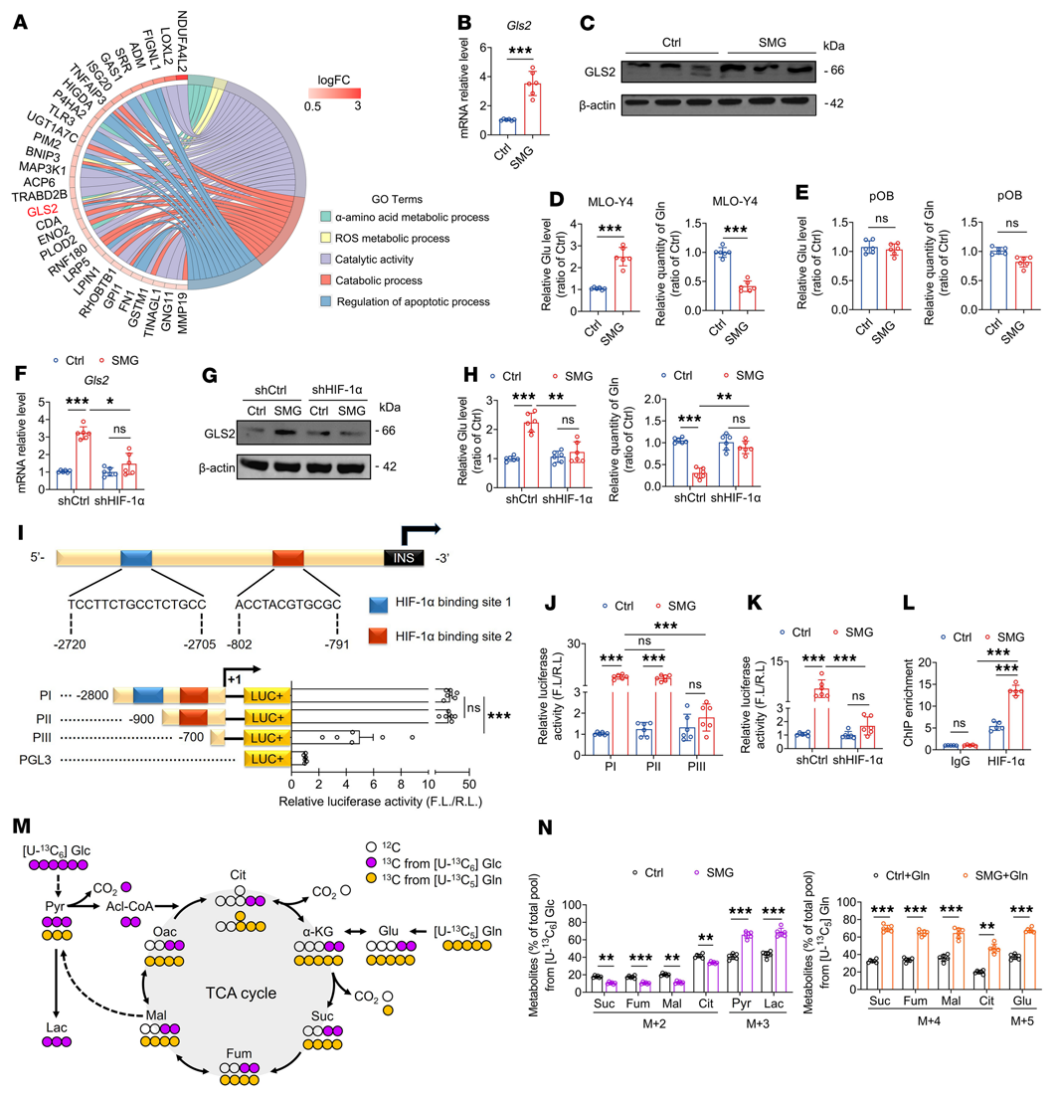
HIF-1α induces substantial GLS2 expression and glutamine addiction in osteocytes with previous unloading. (**A**) GO chord graph showing that GLS2 is a significantly upregulated gene in SMG-exposed MLO-Y4 cells. *n* = 3 per group. (**B** and **C**) qRT-PCR and Western blotting assays of GLS2 expression. *n* = 6 per group. (**D** and **E**) Intracellular glutamate and glutamine concentrations in normal and SMG-exposed MLO-Y4 cells and primary osteoblasts. *n* = 6 per group. (**F**–**H**) qRT-PCR and Western blotting assays of GLS2 expression and assays for intracellular glutamate and glutamine concentrations in normal and SMG-exposed MLO-Y4 cells infected with shHIF-1α lentivirus. *n* = 6 per group. (**I**) Schematic representation of the GLS2 promoter region. Relative luciferase activities of PI, PII, and PIII promoter regions are indicated by bar graphs. PGL3 is the negative control. *n* = 6 per group. (**J**) Relative luciferase activity of PI, PII, and PIII. *n* = 6 per group. (**K**) Relative luciferase activity in normal and SMG-exposed MLO-Y4 cells infected with shHIF-1α lentivirus. *n* = 6 per group. (**L**) ChIP assays demonstrating HIF-1α enrichment on the GLS2 promoter in normal and SMG-exposed MLO-Y4 cells. *n* = 5 per group. (**M**) Schematic drawing of metabolite flux from glucose and glutamine metabolism in the TCA cycle. Purple and orange circles represent ^13^C atoms derived from [U-^13^C]glucose and [U-^13^C]glutamine, respectively. Open circles represent ^12^C atoms. Dashed lines indicate multistep atomic transitions. Acl-CoA, acetyl-CoA; Cit, citrate; Fum, fumarate; Glc, glucose; Gln, glutamine; Glu, glutamate; α-KG, α-ketoglutarate; Lac, lactate; Mal, malate; Oac, oxaloacetate; Pyr, pyruvate; Suc, succinate. (**N**) Contribution of glucose and glutamine carbon to TCA cycle intermediates as measured by mole percent enrichment from [U-^13^C]glucose (M+2) and [U-^13^C]glutamine (M+4) after culturing of MLO-Y4 cells with isotopes for 12 and 6 hours, respectively. *n* = 6 per group. Graphs represent mean ± SD. (**B**, **D**, **E**, and **N**) **P < 0.01, ***P < 0.001 by Student’s *t* test. (**F**, **H**, **J**, **K**, and **L**) *P < 0.05, **P < 0.01, ***P < 0.001 by 2-way ANOVA with Bonferroni’s post-test.

GO analysis based on RNA-Seq revealed that GLS2, a key enzyme that catalyzes the hydrolysis of glutamine to glutamate, was also significantly upregulated in 5 GO terms related to metabolic processes in SMG-exposed MLO-Y4 cells (Figure 5A). Our qRT-PCR and Western blotting results confirmed the upregulation of GLS2, and ELISA assays also revealed an increase in intracellular concentrations of glutamate and a decrease in glutamine concentrations in SMG-exposed MLO-Y4 cells (Figure 5, B–D). In contrast, no significant difference was observed in the glutamine or glutamate levels between the SMG-exposed and control primary osteoblasts (Figure 5E). SMG-induced increase in GLS2 expression and intracellular glutamate levels and decrease in the glutamine concentrations were blunted after HIF-1α silencing in MLO-Y4 cells (Figure 5, F–H). Furthermore, on the basis of transcription factor prediction, we established that HIF-1α in osteocytes has potential binding sites on the GLS2 promoter (Figure 5I). Thus, to identify the promoter sequence recognized by HIF-1α, we truncated the GLS2 promoter region (the sequence from –2800 to +1) into 3 successively smaller fragments, and found that the PIII promoter region (–802 to –791 bp), although not the PII region (–2720 to –2705 bp), is the binding site recognized by HIF-1α (Figure 5I). The promoter activities of all 3 shaved fragments are shown in Figure 5J. We found that luciferase activity was significantly inhibited in SMG-exposed MLO-Y4 cells subjected to HIF-1α blockade (Figure 5K). The chromatin immunoprecipitation (ChIP) results indicate that the enrichment of HIF-1α on the GLS2 promoter was significantly increased in MLO-Y4 cells after SMG exposure (Figure 5L). To further characterize the metabolic routes, we cultured MLO-Y4 cells with [U-^13^C]glucose or [U-^13^C]glutamine, and examined the carbon source as demonstrated by the atom transition map as shown in Figure 5M. SMG exposure resulted in a significant increase in pyruvate and lactate with [U-^13^C]glucose (M+3) and decrease in TCA intermediates, including succinate, fumarate, malate, and citrate labeled with [U-^13^C]glucose (M+2) (Figure 5N). In contrast, [U-^13^C]glutamine (M+4), which contributes to the TCA metabolites, was significantly increased following SMG exposure (Figure 5N), indicating that SMG suppresses glucose oxidation and activates anaplerosis from glutamine oxidation in the TCA cycle. Together, our results revealed that HIF-1α transcriptionally induces GLS2 activation and thereby exhausts endogenous glutamine in osteocytes with previous unloading, thus highlighting the necessity of exogenous glutamine supplementation.

### Glutamine oxidation restores the mechanosensitivity in osteocytes with previous unloading.

**Figure 6:**
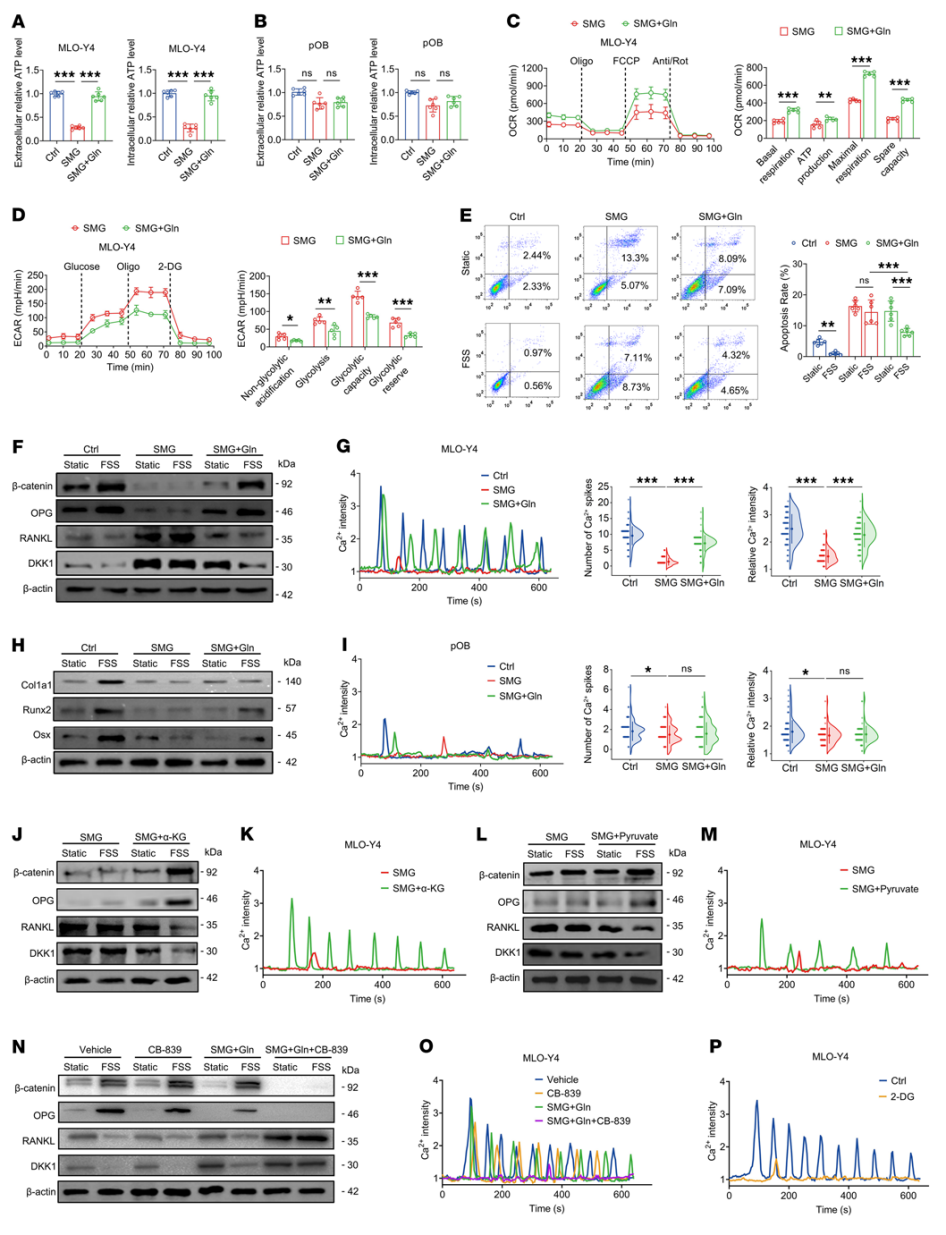
Glutamine oxidation restores mechanosensitivity in osteocytes with previous unloading. (**A** and **B**) Effects of glutamine supplementation on intracellular and extracellular ATP concentration assays in SMG-exposed MLO-Y4 cells and primary osteoblasts, respectively. *n* = 6 per group. (**C** and **D**) Effects of glutamine supplementation on energy metabolism via Seahorse assays to monitor cellular OCR and ECAR in SMG-exposed MLO-Y4 cells. *n* = 5 per group. (**E**–**G**) Effects of glutamine supplementation on intracellular Ca^2+^ dynamics (*n* = 90 cells for Ctrl, *n* = 80 for SMG, *n* = 95 for SMG+Gln), osteocyte-related protein expression, and cell apoptosis (*n* = 6 per group) in response to FSS (2 Pa) in SMG-exposed MLO-Y4 cells. (**H** and **I**) Effects of glutamine supplementation on Ca^2+^ dynamics (*n* = 90 per group) and osteoblast-related protein expression in response to FSS (2 Pa) in SMG-exposed primary osteoblasts. (**J** and **K**) Effects of α-KG supplementation on Ca^2+^ dynamics and osteocyte-related protein expression in response to FSS stimulation in SMG-exposed MLO-Y4 cells. (**L** and **M**) Effects of pyruvate supplementation on Ca^2+^ dynamics and osteocyte-related protein expression in response to FSS stimulation in SMG-exposed MLO-Y4 cells. (**N** and **O**) Effects of the glutaminase inhibitor CB-839 on Ca^2+^ dynamics and osteocyte-related protein expression in response to FSS stimulation in normal MLO-Y4 cells and SMG-exposed MLO-Y4 cells supplemented with glutamine. (**P**) Effects of 2-DG (blocking the conversion of glucose to pyruvate) on Ca^2+^ dynamics in response to FSS stimulation in normal MLO-Y4 cells. Graphs represent mean ± SD. (**C** and **D**) *P < 0.05, **P < 0.01, ***P < 0.001 by Student’s *t* test. (**A**, **B**, **G**, and **I**) *P < 0.05, ***P < 0.001 by 1-way ANOVA with Bonferroni’s post-test. (**E**) **P < 0.01, ***P < 0.001 by 3-way ANOVA with Bonferroni’s post-test.

We went on to examine the effects of glutamine supplementation on energy metabolism and mechanoresponse in SMG-exposed osteocytes and osteoblasts. Provision of glutamine induced significant increases in the concentrations of both extracellular and intracellular ATP in SMG-exposed MLO-Y4 cells, although not in SMG-exposed primary osteoblasts (Figure 6, A and B). Seahorse assays revealed that glutamine supplementation increased the OCR levels and decreased the ECAR levels in SMG-exposed MLO-Y4 cells (Figure 6, C and D). Moreover, FSS had no significant effects on cellular viability or the expression of β-catenin, OPG, RANKL, or DKK1 in SMG-exposed MLO-Y4 cells (Figure 6, E and F, and Supplemental Figure 10). However, in SMG-exposed osteocytes treated with glutamine, FSS induced a significant reduction in the expression of RANKL/OPG and DKK1, and increase in the β-catenin expression and cell viability. Moreover, glutamine induced a significant enhancement of Ca^2+^ oscillatory response to FSS stimulation in SMG-exposed MLO-Y4 cells (Figure 6G), which was almost equivalent to the Ca^2+^ oscillation profile characterizing the response to FSS in normal osteocytes. The positive effects of glutamine on osteocytic Ca^2+^ dynamics were confirmed in tibiae of the HU mice in situ (Supplemental Figure 12). However, glutamine supplementation had no obvious influence on cellular differentiation or mineralization in SMG-exposed osteoblasts in response to FSS stimulation (Figure 6H and Supplemental Figure 11). Glutamine also had no discernible effects on intracellular Ca^2+^ signaling either in Ca^2+^ spike number or intensity in response to FSS in SMG-exposed primary osteoblasts (Figure 6I). We also found that α-KG treatment induced a significant enhancement of Ca^2+^ oscillatory dynamics and β-catenin expression and a significant reduction in the expression of RANKL/OPG and DKK1 in response to FSS in SMG-exposed MLO-Y4 cells (Figure 6, J and K). We further found that pyruvate treatment induced an increase in Ca^2+^ oscillatory dynamics and alteration of osteocyte-related protein expression in response to FSS in SMG-exposed osteocytes (Figure 6, L and M), although pyruvate induced less strong enhancement of mechanoresponse than did α-KG, which was potentially associated with PDK1-mediated inhibition of PDH activity. Furthermore, we evaluated the effects of blocking glutamine and pyruvate metabolism on osteocytic response to FSS stimulation in MLO-Y4 cells. We found that the glutaminase inhibitor CB-839 had no observable effects on mechanoresponse in control MLO-Y4 cells; nevertheless, CB-839 significantly suppressed glutamine-mediated recovery in mechanoresponse in SMG-exposed MLO-Y4 cells (Figure 6, N and O). Moreover, treatment with 2-deoxyglucose (2-DG), a reagent that blocks the conversion of glucose to pyruvate, induced a significant decrease in mechanoresponse in control MLO-Y4 cells (Figure 6P and Supplemental Figure 13). Together, our findings provide convincing evidence that unloading induces a metabolic phenotype transition from glucose dependency to glutamine dependency, and exogenous glutamine restores the mechanosensitivity to reloading in osteocytes previously exposed to unloading.

### Glutamine supplementation enhances bone mechanosensitivity to reloading in mice previously exposed to HU via osteocyte-mediated regulation of osteoblasts and osteoclasts.

**Figure 7:**
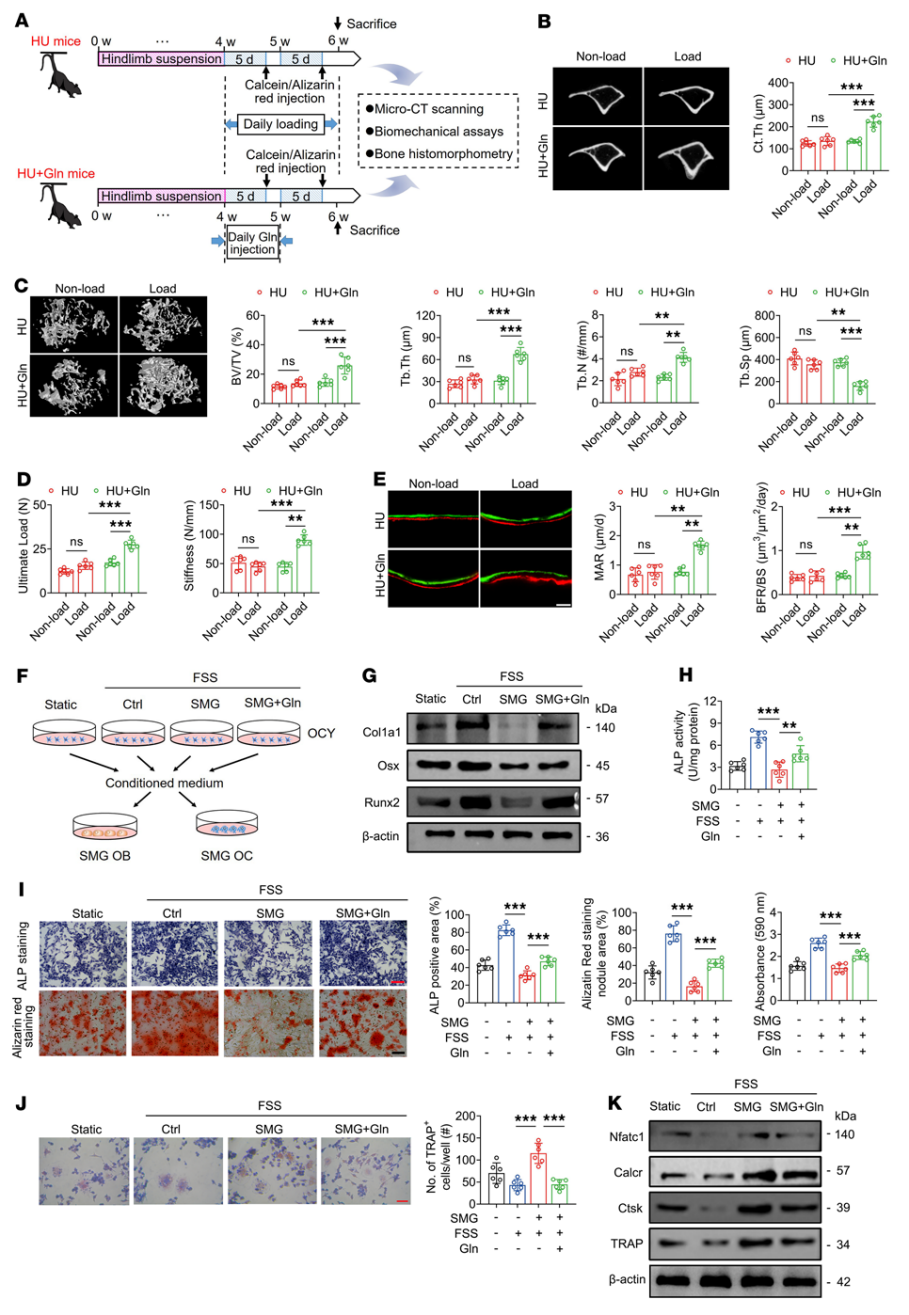
Glutamine supplementation enhances bone mechanosensitivity to reloading in mice previously exposed to HU via osteocyte-mediated regulation of osteoblasts and osteoclasts. (**A**) Experimental protocol of 4-week tail suspension to establish the HU model, subsequent glutamine supplementation (daily injection at 673 mg/kg body weight via tail vein for 1 week), and mechanical reloading with uniaxial cyclic compression (1,200 cycles/day for 2 weeks) in mice. (**B** and **C**) Representative micro-CT images showing proximal tibial trabecular bone architecture and cortical bone thickness, and the corresponding quantitative data. *n* = 6 per group. (**D**) Three-point bending tests for assessing whole-bone mechanical properties. *n* = 6 per group. (**E**) Dynamic bone histomorphometry using calcein and alizarin red double labeling. *n* = 6 per group. (**F**) Schematic representation of conditioned medium collection from SMG-exposed MLO-Y4 cells treated with or without glutamine in response to FSS stimulation (2 Pa for 3 hours), and its incubation in SMG-exposed primary osteoblasts and RAW264.7 osteoclast precursor cells. (**G**–**I**) Survival and differentiation assays of SMG-exposed primary osteoblasts treated with conditioned medium collected from SMG-exposed MLO-Y4 osteocytic cells with glutamine supplementation in response to FSS stimulation. (**G**) Western blotting assays of protein expression of Col1a1, Runx2, and Osx in primary osteoblasts. (**H** and **I**) ALP activity, ALP staining, and alizarin red staining assays in primary osteoblasts. *n* = 6 per group. (**J** and **K**) Osteoclastogenesis assays in SMG-exposed RAW264.7 cells (pre-osteoclasts) treated with conditioned medium collected from SMG-exposed MLO-Y4 cells with glutamine supplementation in response to FSS stimulation. (**J**) TRAP staining to quantify the formation of osteoclasts. *n* = 6 per group. (**K**) Western blotting analyses of the expression of osteoclast-related markers in RAW264.7 cells, including TRAP, cathepsin K, NFATc1, and calcitonin receptor. Graphs represent mean ± SD. **P < 0.01, ***P < 0.001 by 2-way ANOVA with Bonferroni’s post-test. Scale bars: **E**, 30 μm; **I** and **J**, 50 μm.

We next examined the effects of glutamine supplementation on bone mechanosensitivity to cyclic compressive reloading (1,200 cycles/day for 2 weeks) in mice previously subjected to tail suspension for 4 weeks (Figure 7A). The blood glutamine levels in the HU plus glutamine (HU+Gln) group were found to be significantly higher than those in the HU group (Supplemental Figure 14). Micro-CT and biomechanical analyses revealed that, following reloading, HU mice that had received glutamine supplementation exhibited significant improvements in tibial cortical bone thickness, trabecular bone mass and architecture, and whole-bone mechanical properties compared with those of unsupplemented HU mice (Figure 7, B–D). Glutamine supplementation significantly increased the number of viable osteocytes, and also reduced the number of dying/dead osteocytes and expression of sclerostin and RANKL in the tibial osteocytes of HU mice subjected to reloading (Supplemental Figure 14). Furthermore, in response to mechanical reloading, glutamine-supplemented HU mice were observed to have a significantly higher number of osteoblasts on trabecular bone surfaces and a higher rate of hind-limb bone formation than unsupplemented HU mice (Figure 7E and Supplemental Figure 14). These results suggest that the administration of exogenous glutamine to HU mice significantly augments the mechanosensitivity of bone to reloading in these mice. We subsequently sought to determine the regulatory role of osteocytes in the glutamine-mediated sensitization to reloading in skeletons with previous unloading. To this end, osteoblasts and osteoclasts that had been exposed to SMG in vitro were incubated with the conditioned medium collected from glutamine-treated SMG-exposed MLO-Y4 cells that had been subjected to FSS reloading for 3 hours (Figure 7F). The conditioned medium from osteocytes in the SMG+Gln+FSS group induced significantly higher osteogenic differentiation and mineralization than that in the SMG+FSS group, according to the alkaline phosphatase (ALP) staining and ALP activity, alizarin red staining, and osteogenic marker (Col1a1, Runx2, and Osx) expression assays (Figure 7, G–I, and Supplemental Figure 15). Moreover, the findings of tartrate-resistant acid phosphatase (TRAP) staining, cytoskeleton immunofluorescence staining, and osteoclast-related gene and protein expression (TRAP, cathepsin K, NFATc1, and calcitonin receptor) assays revealed that exposure to the conditioned medium collected from MLO-Y4 cells in the SMG+Gln+FSS group resulted in a significant suppression of osteoclast differentiation and maturation compared with that collected from cells in the SMG+FSS group (Figure 7, J and K, and Supplemental Figure 15). However, we detected no significant difference between the HU and HU+Gln groups with respect to body weight and food intake (Supplemental Figure 14). Collectively, our results thus indicate that the glutamine-induced enhancement of mechanosensitivity to reloading in skeletons previously exposed to unloading is primarily associated with the mechanical sensitization of osteocytes and the osteocyte-mediated regulation of osteoblasts and osteoclasts.

### Inhibition of glycolysis by blockade of PDK1 sensitizes the mechanoresponse to reloading in bone and osteocytes exposed to previous unloading.

**Figure 8:**
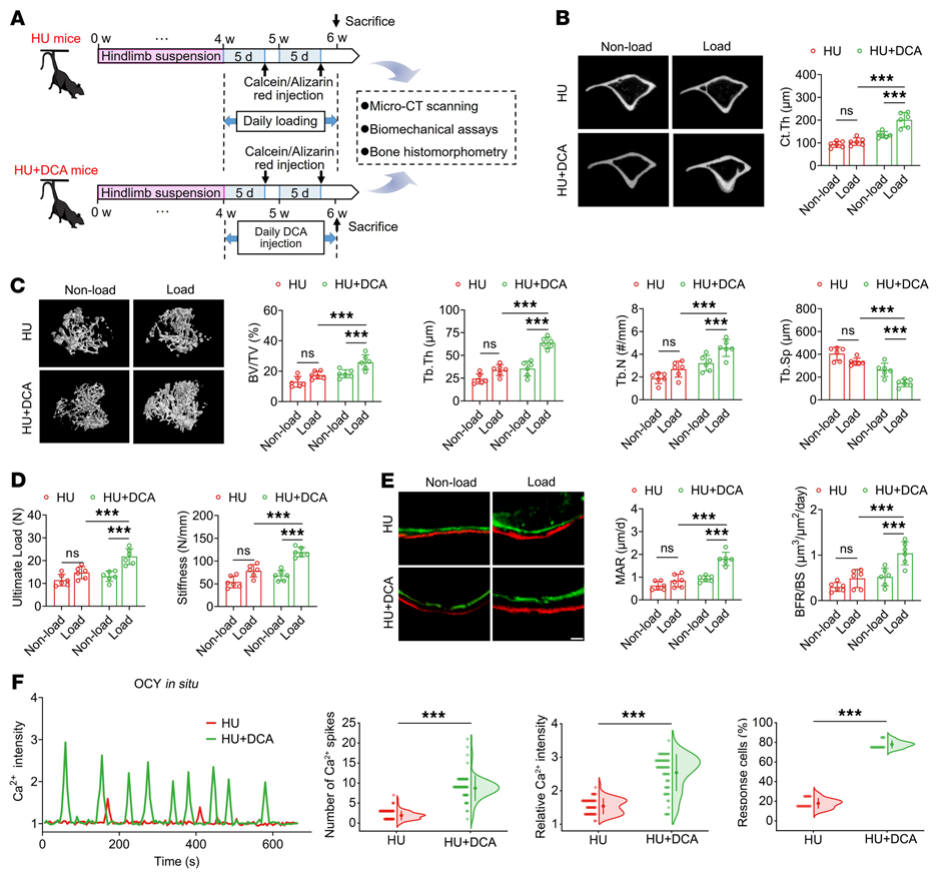
Inhibition of glycolysis by blocking of PDK1 sensitizes the mechanoresponse to reloading in bone and osteocytes exposed to previous unloading. (**A**) Experimental protocol of 4-week tail suspension to establish the HU model, subsequent PDK1 antagonist treatment (daily 200 mg/kg body weight for 2 weeks), and mechanical reloading with uniaxial cyclic compression (1,200 cycles/day for 2 weeks) in mice. (**B** and **C**) Representative micro-CT images showing proximal tibial trabecular bone architecture and cortical bone thickness, and the corresponding quantitative data. *n* = 6 per group. (**D**) Three-point bending tests for assessing whole-bone mechanical properties. *n* = 6 per group. (**E**) Dynamic bone histomorphometry using calcein and alizarin red double labeling, and the corresponding quantitative data. *n* = 6 per group. (**F**) Comparison of intracellular Ca^2+^ signaling in tibial osteocytes in situ in tail-suspended mice in the presence and absence of PDK1 antagonist treatment in response to subsequent uniaxial cyclic compressive loading at 9 N, and the corresponding quantitative data. *n* = 72 per group. Graphs represent mean ± SD. (**B**–**E**) ***P < 0.001 by 2-way ANOVA with Bonferroni’s post-test. (**F**) ***P < 0.001 by Student’s *t* test. Scale bar: **E**, 30 μm.

We then studied the effects of the inhibition of glycolysis by administration of the PDK1 antagonist dichloroacetate (DCA) on bone mechanosensitivity to reloading with cyclic compression in tail-suspended mice (Figure 8A). DCA administration resulted in a marked increase in cortical bone thickness and trabecular bone mass in the tibia of tail-suspended mice in response to cyclic reloading, according to the micro-CT results (Figure 8, B and C). Similarly, the findings of 3-point bending tests revealed that DCA induced a significant improvement in tibial mechanical strength in HU mice subjected to reloading (Figure 8D). The results of dynamic bone histomorphometry indicated that the rate of new bone formation in the tibiae of HU mice subjected to mechanical reloading was significantly enhanced when the mice were treated with the PDK1 antagonist (Figure 8E). No significant difference was observed between the HU and HU+DCA groups with respect to body weight or food intake (Supplemental Figure 16). Moreover, compared with HU mice that did not receive the DCA treatment, DCA-treated HU mice were found to be characterized by a significantly higher intensity and frequency of osteocytic Ca^2+^ oscillations in situ in response to cyclic compressive loading, which was similar to the osteocytic Ca^2+^ oscillatory profile observed in normal mice (Figure 8F). Our in vitro results revealed that in response to FSS stimulation, the SMG-exposed MLO-Y4 osteocytic cells that has been subjected to PDK1 silencing (the knockdown efficiency is shown in Supplemental Figure 8) had higher cell viability and gene and protein expression of β-catenin and OPG and lower RANKL and DKK1 than the SMG-exposed nontransfected cells (Supplemental Figure 17). However, blockade of PDK1 was found to have no discernible effects on cellular differentiation in vitro in SMG-exposed primary osteoblasts following FSS stimulation, as evidenced by the results obtained for ALP staining, ALP activity, alizarin red staining, and qRT-PCR and Western blotting assays of Col1a1, Osx, and Runx2 expression (Supplemental Figure 18). Our results suggest that blockade of PDK1 sensitizes the mechanosensitivity to reloading in bone exposed to previous unloading by primarily modulating osteocytes.

## Discussion

The sluggish and insufficient recovery of bone mass during reambulation after prolonged disuse (e.g., long-term therapeutic bed rest, limb immobilization, and spaceflight) represents an unresolved medical challenge (27, 28). In this study, we provide the first direct evidence, to our knowledge, of significantly reduced bone mechanosensitivity to reloading after disuse/unloading, which occurs as a consequence of the alteration of osteocytes and their Ca^2+^ oscillatory dynamics. We also found that the disuse/unloading-induced compromised osteocytic Ca^2+^ response to reloading is associated with a HIF-1α/PDK1–mediated specific increase in glycolysis. HIF-1α was also found to transcriptionally induce substantial GLS2 expression and subsequent endogenous glutamine exhaustion. Blockade of PDK1 or supplementation with glutamine restores mechanosensitivity to reloading in bone tissues exposed to previous unloading by fueling the TCA cycle and subsequent ATP production in osteocytes. This study has accordingly enabled us to identify novel alternative approaches that could be adopted to accelerate bone recovery in individuals after long-duration disuse/unloading.

Although in the clinical setting, those who have experienced long-term disuse/microgravity are encouraged to undertake progressive weight-bearing exercise, there are certain aspects of bone morphology and quality that fail to return to the original levels, even after reloading stimulation (2). Here, using mice that had previously been subjected to HU, we established a constrained unilateral tibial loading model incorporating a controllable loading regime to assess the skeletal benefits gained from reloading compared with the untreated contralateral tibia (29). We found that a 2-week course of mechanical loading was not nearly sufficient to compensate for the loss of both trabecular and cortical bone induced by 4-week tail suspension in these mice. In particular, we detected a negligible increase (<10%) in cortical bone thickness and whole-bone mechanical strength in response to reloading, indicating that mechanical loading is insufficient to induce a rapid recovery of bone mass and strength following disuse/unloading. Further bone metabolism analyses revealed that reloading induces minor changes in osteocyte activity, bone formation, and bone resorption in tail-suspended mice. To the best of our knowledge, this study is the first to reveal the compromised response of bone mechanosensitivity to reloading after disuse/microgravity.

**Figure 9:**
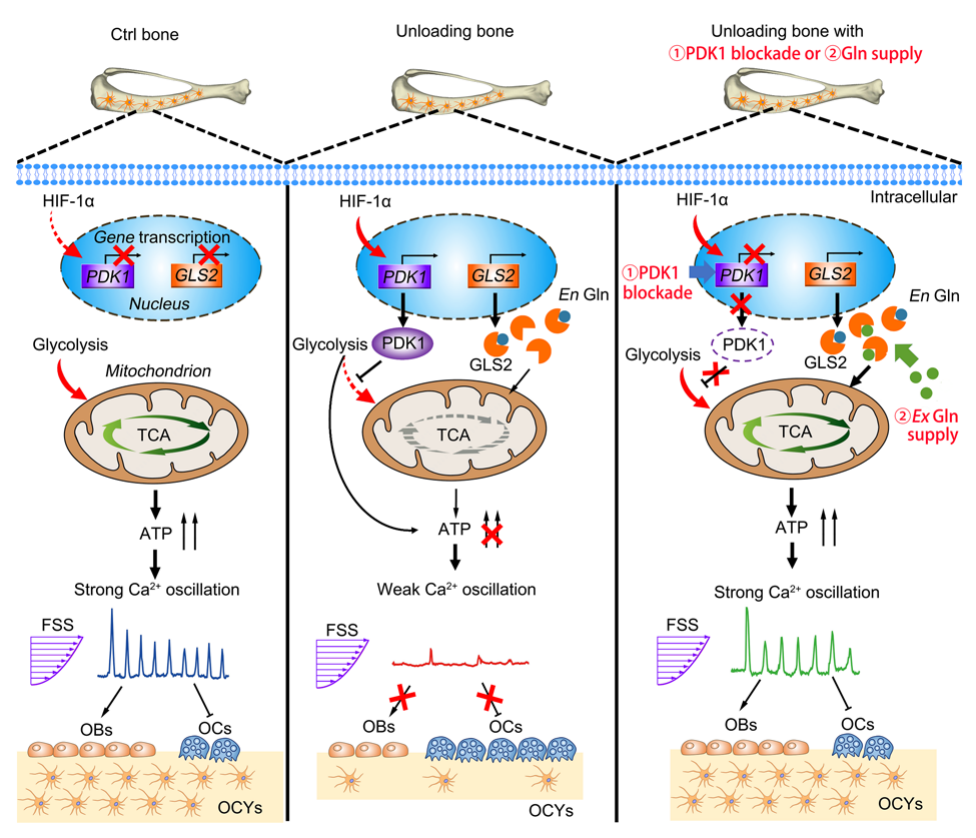
Schematic drawing of the mechanism by which disuse/unloading induces the deterioration of bone mechanosensitivity and inhibition of glycolysis by blockade of PDK1 or glutamine supplementation accelerates bone recovery after long-duration disuse/unloading.

The mechanism by which disuse/unloading induces the deterioration of bone mechanosensitivity is schematically summarized in Figure 9. The extensive osteocyte network embedded in the bone matrix is morphologically similar to the neuronal network, and has been demonstrated to have a higher capacity in sensing and memorizing external mechanical cues than other bone cell types (21, 30, 31). Using novel multiscale cellular Ca^2+^ imaging technologies, we detected robust Ca^2+^ oscillations in normal osteocytes both in vitro and in situ, whereas only weak Ca^2+^ responses were observed in osteocytes that had previously been exposed to unloading. This unique Ca^2+^ oscillation with repetitive robust Ca^2+^ spikes signifies a higher efficiency and specificity in the regulation of gene expression than other Ca^2+^ signaling profiles (e.g., a sustained Ca^2+^ rise or constant Ca^2+^ signals) (32), and also mediates cytokine secretion and cell contraction (33, 34). In contrast, unloading-exposed osteoblasts exhibited no difference in Ca^2+^ response to reloading compared with normal osteoblasts. Accordingly, our findings provide evidence to indicate that the disuse/microgravity-induced reduction in bone mechanosensitivity is primarily attributable to the alteration of osteocytes but not osteoblasts, and consequently, the manipulation of osteocyte Ca^2+^ dynamics may represent an effective approach for restoring the mechanical responsiveness of bone following disuse/unloading.

In this study, we also demonstrated that ATP played a key role in the mechanically induced Ca^2+^ oscillations in osteocytes both in vitro and in situ. More importantly, we detected a markedly reduced production of ATP in SMG-exposed osteocytes, but not in osteoblasts, and suspect that this may be a key factor contributing to the reduced bone mechanosensitivity following disuse/unloading. Extracellular ATP activates G protein–coupled P_2_Y receptors on plasma membrane that in turn stimulate phospholipase C–mediated inositol 1,4,5-triphosphate (IP_3_) generation and subsequent Ca^2+^ release from the ER Ca^2+^ stores, and thereby induces dynamic changes in cytosolic Ca^2+^ concentrations (35). Moreover, it has been established that the propagation of intercellular Ca^2+^ waves in the bone cell network is dependent on the diffusion of extracellular ATP (21, 36). Thus, the reduction in ATP production induced by unloading disrupts the spatiotemporal Ca^2+^ dynamics of osteocytes in response to reloading via both autocrine and paracrine mechanisms. Furthermore, mechanically induced Ca^2+^ oscillations in normal osteocytes were remarkably suppressed following the inhibition of mitochondrial ATP generation with antagonists, which was similar to the Ca^2+^ profiles observed in unloading-exposed osteocytes. Thus, favorably modifying the bioenergetics of osteocytes exposed to unloading is considered a key strategy for enhancing the Ca^2+^ response of these cells and the mechanosensitivity of bone to reloading.

Our transcriptome screens revealed substantial changes in the glucose metabolism of osteocytes subjected to unloading, characterized by a distinct metabolic switch from oxidative phosphorylation to glycolysis, which is accordingly deemed to be indicative of a significant reduction in bioenergetic efficiency (37). We also observed a high enrichment in hypoxia-related biological processes and the oxygen homeostasis–related HIF-1α pathway in SMG-exposed osteocytes. This is consistent with the findings of several previous studies that have reported similar patterns of hypoxia and HIF-1α activation in immune and nerve cells following exposure to microgravity (38, 39). Moreover, pharmacological activation of HIF-1α is sufficient to block energy metabolism and mechanoresponse in normal osteocytes, which is similar to the SMG effects. Under hypoxic conditions, HIF-1α is stabilized and translocated into the nucleus, wherein it interacts with the hypoxia response element in the promoter regions of multiple genes (40). We also observed a specific increase in the expression of PDK1, a gatekeeper enzyme associated with glucose metabolic reprogramming, and found that blockade of HIF-1α inhibited the SMG-induced increase in PDK1 expression and reduction in PDH activity. Similarly, the findings of several previous studies have indicated that HIF-1α mediates the transcriptional regulation of PDK1 under hypoxic conditions (41, 42). Our findings provide strong evidence to indicate that the observed reduction in mechanosensitivity following unloading is the consequence of a HIF-1α/PDK1–mediated increase in osteocyte glycolysis. Importantly in this regard, we demonstrated that injection of a PDK1 inhibitor restored the mechanosensitivity of bone following unloading, thereby highlighting the importance of modulating osteocyte glycolysis and bioenergetics.

Cells with heightened levels of glycolytic respiration tend to be highly dependent on glutamine (43), and glutamine-driven oxidative phosphorylation is also an important source of ATP in mammalian cells under both normoxic and hypoxic conditions (44). We found that HIF-1α also transcriptionally induces substantial GLS2 expression in SMG-exposed osteocytes, thereby activating glutamine catabolic processes. As the major site of glutamine synthesis, the musculoskeletal system is characterized by rapid and substantial glutamine consumption during catabolic stress (e.g., surgery and trauma) (45). Similarly, we detected an exhaustion of glutamine and an increase in glutamine oxidation flux in osteocytes following SMG exposure, indicating that osteocytes may gain a benefit from exogenous glutamine supplementation by fueling the TCA cycle. Indeed, we discovered that glutamine or α-KG supplementation promoted the recovery of the mechanosensitivity of bone and osteocytes (although not osteoblasts) following unloading, which was shown to be equivalent to the effects of a PDK1 antagonist. Glutamine-mediated recovery in mechanoresponse in unloading-exposed osteocytes was abolished after blockade of glutamine metabolism using CB-839. Moreover, we discovered that the conditioned medium obtained from SMG-exposed osteocytes treated with glutamine can contribute to the regulation of both osteoblasts and osteoclasts, thereby further emphasizing the central role of osteocytes in bone mechanoresponse after unloading.

Glutamine is a non-essential amino acid, abundant dietary sources of which include milk, meat, and nuts. From a therapeutic perspective, this amino acid is a considerably safer and more readily available agent than PDK1 inhibitors that have been established to have potential adverse side effects and cytotoxicity. Glutamine is also one of the most prominent amino acid supplements taken by bodybuilders and fitness enthusiasts (46, 47). Moreover, glutamine supplementation is typically beneficial to patients with minimal energy reserves or catabolic stress, such as postoperative/elderly patients and low-birthweight infants (47). The findings of recent studies have revealed that targeting glutamine metabolism is an effective approach for treating numerous diseases, including intestinal ischemia/reperfusion, alopecia, and various complications during cancer radiotherapy and chemotherapy (48–50). Thus, we believe that glutamine supplementation would have promising clinical applications with respect to accelerating bone recovery following reambulation.

In conclusion, this study not only provides fundamental insights into the deterioration of bone mechanosensitivity following disuse/unloading, which is dependent on abnormal osteocytic Ca^2+^ oscillatory dynamics primarily induced by a metabolic switch from oxidative phosphorylation to glycolysis, but also more importantly offers a proof of principle that weight-bearing activity coupled with nutritional or pharmacological modulation of ATP production (i.e., glutamine supplementation or PDK1 inhibition) may represent a novel therapeutic strategy for accelerating bone recovery after long-duration disuse/unloading.

## Methods

Further information can be found in Supplemental Methods.

### Animal models.

All animal experiments were approved by the Institutional Animal Care and Use Committee of the Fourth Military Medical University. The care and protection of experimental animals were in accordance with the guidelines and regulations on animals of the US National Institutes of Health and the Chinese National Institute of Health. Three-month-old male C57BL/6J mice were obtained from the Animal Center of the Fourth Military Medical University. Animals were housed under a controlled temperature of 24°C ± 1°C with a 12-hour light/12-hour dark cycle. To establish the hind-limb unloading (HU) model, mice were maintained in an approximately 30° head-down-tilt position by tail suspension using a custom-designed Plexiglas cage for 4 weeks. Then, the right tibiae of mice were subjected to daily uniaxial cyclic compressive loading for 2 weeks (5 d/wk) using a custom-designed mechanical loading system as described previously (29, 51). The contralateral left tibiae were not loaded and were used as controls. To examine the role of glutamine supplementation and PDK1 inhibition in rescuing bone’s sensitivity to reloading in HU mice, glutamine (673 mg/kg body weight) was injected daily via tail vein for 1 consecutive week (52), and the PDK1 inhibitor (DCA; 200 mg/kg body weight) was intragastrically administered every day for 2 weeks (53) from the first reloading day. Mice in the control group were injected with the same dose of saline. All mice were intraperitoneally injected with alizarin red (30 mg/kg; MilliporeSigma) and calcein (30 mg/kg; MilliporeSigma) 9 days and 2 days before euthanasia, respectively.

### In vivo mechanical loading.

A physiological-related cyclic loading with peak tensile strain of 1,500 με was generated on the anteromedial surface of the mouse tibia in the control, HU, HU+Gln, and HU+DCA groups. The strain measurements were conducted on a separate set of male C57BL/6J mice (*n* = 6 per group; Animal Center of the Fourth Military Medical University). A strain gauge (EA-06-015DJ-120, Measurements Group Inc.) was glued to a relatively flat anteromedial surface of the tibia. A preload with 1 N was given to immobilize the tibia, and then compressive loading was applied at 0.2 mm/s until reaching 1,500 με strain. The load magnitudes of 9.0 ± 0.6 N, 7.5 ± 0.5 N, 7.7 ± 0.3 N, and 7.9 ± 0.4 N corresponding to 1,500 με strain on tibial surfaces were obtained in the mice of the control, HU, HU+Gln, and HU+DCA groups, respectively. The accuracy of strain gauge measurements was verified using micro-CT–based finite element analysis (Supplemental Figure 1). For the application of daily cyclic loading, mice were anesthetized via intraperitoneal injection of 50 mg/kg sodium pentobarbital, and then transferred to a custom-designed mechanical loading system (Supplemental Figure 1). A preload of 1 N was given to immobilize the hind limb, and then cyclic compressive loading was applied with a ramp loading waveform at 4 Hz with 1,200 cycles/day.

### In situ osteocyte Ca^2+^ imaging in bone under cyclic compressive loading.

Mice after tail suspension were sacrificed, and bilateral intact tibiae were immediately dissected. After gentle removal of muscles under sterile conditions, tibiae were immersed within α-MEM containing 5% FBS, 5% calf serum (CS), and 1% penicillin/streptomycin (P/S) for 2 hours. Then, tibiae were incubated with 5 μM Calbryte-520 AM (AAT Bioques) dissolved in DMSO with 0.04% pluronic acid F-127 (10% in water) in phenol red–free α-MEM for 1 hour. After washing, samples were transferred to a custom-designed mechanical loading system (Supplemental Figure 1). The anteromedial surface with prominent tensile strains under compressive loading was selected as the imaging region using a confocal microscope (Fluoview FV3000, Olympus, Japan) with ×10 objective and 488 nm laser excitation. Confocal time-lapse images were synchronized with a rest-inserted loading protocol to avoid the drift of confocal objective focus during dynamic loading. A preload of 1 N followed by 124 cycles of 9.0 ± 0.6 N, 7.5 ± 0.5 N, 7.7 ± 0.3 N, and 7.9 ± 0.4 N peak load (corresponding to 1,500 με tensile strain) was applied on tibiae in mice of the control, HU, HU+Gln, and HU+DCA groups, respectively. A dwell time of 4 seconds was applied between each cycle. Fluorescence images were captured at 1.1 s/frame during the dwell time after each loading cycle for a total of 129 frames including 5-frame baseline recording before loading. The percentage of responsive cells, average Ca^2+^ spike number, intensity of Ca^2+^ spikes, and Ca^2+^ spike initiation time were quantified using Fluoview FV31S-SW software.

### Cell culture.

MLO-Y4 cells, a cell line of osteocytes obtained from Lynda F. Bonewald (Department of Oral Biology, University of Missouri–Kansas City, Kansas City, Missouri, USA), were cultured in a collagen-coated dish (rat tail collagen type I, BD Biosciences) in α-MEM containing 5% FBS, 5% CS, and 1% P/S. RAW264.7 cells (Cell Bank of Chinese Academy of Sciences, Shanghai, China) were cultured in DMEM supplemented with 10% FBS, 1% P/S, and 4.5 g/L glucose. Cells were then exposed to 50 ng/ml RANKL (PeproTech) to induce osteoclastogenesis. Primary osteoblasts were obtained from mouse calvaria by the triple collagenase/dispase II digestion method (54). Cells were then incubated in α-MEM supplemented with 10% FBS and 1% P/S. To induce osteogenic differentiation, cells were transferred to osteoblast differentiating medium (α-MEM containing 10% FBS, 50 μg/mL ascorbic acid, and 4 mM β-glycerophosphate). Primary osteocyte isolation was performed with a protocol as described previously (55, 56). In brief, femoral and tibial samples of 3-month-old male C57BL/6J mice were aseptically dissected. After flushing with PBS to remove bone marrow, the midshaft was then cut into approximately 1-mm^3^ bone fragments. Samples were subjected to serial digestion with collagenase II (1 mg/mL) and demineralization with EDTA (5 mM) alternately. Isolated primary osteocytes from digest fractions 8–9 were directly plated and cultured in a collagen-coated dish filled with α-MEM containing 5% FBS, 5% CS, and 1% P/S.

### The SMG cell model and fluid flow stimulation.

A 2-dimensional rotating wall vessel bioreactor (developed by China Astronaut Research and Training Center) was used to establish the in vitro SMG model (57). In brief, cells were grown in a T-25 cell culture flask until reaching 40% confluence. The flask was fully filled with the cell culture medium to clear away any air bubble, and then mounted into the rotating bioreactor at 37°C and 5% CO_2_. The vessels were rotated around the horizontal axis at 24 rpm for 48 hours. Cells in the control group were incubated for 48 hours at 37°C in the stationary position (normal gravity). Then, after trypsinization, cell suspensions with approximately 1 × 10^5^ cells/mL were seeded onto the surface of type I rat tail collagen–coated glass slides at 37°C for 24 hours before fluid flow stimulation. The steady or oscillating (1 Hz) laminar fluid flow at 2 Pa was applied on the cell surface for 3 hours at 37°C through the chamber driven by a peristaltic pump. Then, cells were collected for the following assays.

### In vitro nutrient supplementations.

After 48-hour SMG exposure using normal cell culture medium, cells in the control and SMG groups were cultured with glutamine-free medium for 3 days. Cells in the SMG+Gln group were treated with glutamine-free medium supplemented with extra 4 mM glutamine (25030081, Gibco), and cells in the SMG+α-KG group were treated with glutamine-free cell culture medium supplemented with 2 mM methyl-α-KG (SML2205, MilliporeSigma). Then, cells were subjected to energy metabolic assays and mechanoresponse experiments. For the pyruvate supplementation experiment, after 48-hour SMG exposure using normal medium, cells in the SMG group were cultured with pyruvate-free medium for 3 days, and cells in the SMG+pyruvate group were incubated with the medium supplemented with extra pyruvate (107360, MilliporeSigma; the final pyruvate concentration was 2 mM) for 3 days.

### In vitro Ca^2+^ imaging under fluid flow stimulation.

After SMG exposure, cells seeded on glass slides were incubated with 2.5 μM Calbryte-520 AM for 1 hour. The slide was then mounted into the parallel plate flow chamber, and transferred to the confocal microscope with a ×20 objective at 488 nm laser excitation. Laminar fluid flow was then applied through the chamber driven by the peristaltic pump. The Ca^2+^ responses of cells were recorded at 3.2 seconds per frame for a total of 200 frames: 20 frames for baseline and 180 frames after the onset of fluid flow. Cells were traced for Ca^2+^ signaling analyses using Fluoview FV31S-SW software. The intensity of Ca^2+^ spikes of each cell was normalized by its corresponding baseline.

### Seahorse-based bioenergetic analysis.

OCR and ECAR were measured using the Seahorse Bioscience XFe24 flux analyzer (Agilent Technologies) according to the manufacturer’s instructions. Briefly, cells were seeded on collagen-coated 24-well Seahorse XFe plates at a density of 5 × 10^4^ cells per well overnight. Then, OCR was determined in basal assay medium supplemented with 1 mM pyruvate, 10 mM glucose, and 2 mM glutamine followed by sequential treatment with 1 μM oligomycin, 2 μM FCCP, and 0.5 μM rotenone/antimycin A. For the ECAR measurement, cells were seeded at a density of 5 × 10^4^ cells per well in glucose-free XF assay medium containing 2 mM glutamine by the sequential addition of 10 mM glucose, 1 μM oligomycin, and 50 mM 2-DG. Measurements were normalized to the cell number in each well at the end of the assay and analyzed using Seahorse Wave software.

### Stable isotope tracing analysis of glycolysis and TCA cycle intermediates.

Cells were seeded in a 6-well plate in triplicate wells and cultured overnight. The culture medium was replaced with α-MEM with 10% FBS supplemented with [U-^13^C_6_]glucose for 12 hours or [U-^13^C_5_]glutamine (>99%; Cambridge Isotope Laboratories) for 6 hours. Cells were then rinsed with 0.9% (wt/vol) saline twice and quenched with 500 mL of –20°C methanol. Then, 200 μL of ice-cold water containing 1 mg of norvaline internal standard (Cambridge Isotope Laboratories) was added into the cell lysates and transferred to fresh sample tubes. Then 500 mL of –20°C chloroform was added into the tubes, and extracts were vortexed for 15 minutes. Samples were centrifuged at 13,680 *g* for 10 minutes at 4°C. The top aqueous layer was collected and evaporated in a vacuum centrifuge at 4°C. Dried intracellular metabolites were kept at –20°C until derivatization for gas chromatography–mass spectrometry analysis. To interpret the labeling patterns, mole percentage enrichment of isotopes was calculated as the percentage of all atoms within the metabolite pool that were labeled.

### RNA-Seq analysis and bioinformatics.

Total RNA was extracted using an RNA isolation kit (TR205, Beijing Tianmo Biotechnology) according to the manufacturer’s protocol. Paired-end libraries were synthesized using the TruSeq RNA sample preparation kit (Illumina). After purification, the mRNA was fragmented into small pieces and the cleaved RNA fragments were converted to cDNA. The products were purified and enriched with PCR to create the final cDNA library. After cluster generation, the libraries were sequenced using an Illumina NovaSeq 6000 platform (Illumina). Fragments per kilobase of exon per million reads mapped (FPKM) was quantified using StringTie and Ballgown. Significantly differentially expressed RNAs with the absolute value of fold change greater than 1.5 and *P* value less than 0.05 were annotated to GO terms and KEGG pathways. The GSEA analysis (http://www.broad.mit.edu/gsea/index.html) was performed based on the known target genes in the Molecular Signatures Database (MSigDB) gene sets. The RNA-Seq data were deposited in the NCBI’s Sequence Read Archive (SRA) under accession number PRJNA865916.

### Statistics.

All data were analyzed for statistical significance using SPSS 26.0. All data were normally distributed and homoscedastic according to the Kolmogorov-Smirnov test and Levene’s test, respectively. *P* value was determined by 2-tailed unpaired Student’s *t* test for comparisons between 2 groups. For comparisons between more than 2 groups, 1-way ANOVA with Bonferroni’s post hoc test was used for 1 condition, and 2-way ANOVA with Bonferroni’s post hoc test was used for 2 conditions. A *P* value less than 0.05 was considered significant. Quantitative data are presented as mean ± SD.

### Study approval.

All animal experiments were approved by the Institutional Animal Care and Use Committee of the Fourth Military Medical University and were in accordance with the guidelines and regulations for the care and protection of animals (approval no. 20180303).

## Author contributions

DJ, XEG, LS, and EL designed research. XL, YY, DW, YD, XS, XH, PL, and JC performed research. XL, YY, ZY, LS, PL, and DJ analyzed data. XL, ZY, LS, and DJ wrote the paper.

## Supplementary Material

Supplemental data

Supplemental tables 1-4

## Version 1 12/13/2022

In-Press Preview

## Version 2 02/01/2023

Electronic publication
