# Supplementary material for: Glucose- and glutamine-dependent bioenergetics sensitize bone mechanoresponse after unloading by modulating osteocyte calcium dynamics
Source: J Clin Invest. 2023 Feb 1;133(3):e164508. doi: 10.1172/JCI164508 (PMC9888392; doi:10.1172/JCI164508)
Supplement: Supplemental data [file jci-133-164508-s125.pdf]

Supplementary Materials for

Glucose- and glutamine-dependent bioenergetics sensitize bone mechanoresponse after unloading by modulating osteocyte calcium dynamics

Xiyu Liu; Zedong Yan; Jing Cai; Dan Wang; Yongqing Yang; Yuanjun Ding; Xi Shao; Xiaoxia Hao; Erping Luo; X. Edward Guo; Peng Luo*; Liangliang Shen*; Da Jing*

*Corresponding author. Email: pengluo@fmmu.edu.cn; bioliangshen@163.com; jingdaasq@126.com

This PDF file includes:

Supplemental Materials and Methods
Supplemental Figures 1 to 18

Supplemental Materials and Methods

Micro-CT scanning and three-point bending tests

Bilateral tibiae were harvested and wrapped in saline-soaked gauze and stored at -20°C, and then scanned using a micro-CT apparatus (SKYSCAN 1278, Bruker BioSpin, Karlsruhe, Germany) with the isotropic resolution of 8 μm, voltage of 55 kV, current of 200 μA, and rotation step of 0.36°. The VG Studio MAX 2.2 software (Volume Graphics, Heidelberg, Germany) was used for 3D model reconstructions and bone morphometric analyses, including trabecular bone volume fraction (BV/TV), trabecular thickness (Tb.Th), trabecular number (Tb.N), trabecular separation (Tb.Sp), and cortical thickness (Ct.Th). The trabecular volume of interest (VOI) was selected from the growth plate of the proximal tibia and extend 1 mm to the distal end, which excluded the primary spongiosa. Cortical bone cross-sections at distances of 37% of the tibial length from its proximal end were selected to analyze the cortical bone thickness. After micro-CT scanning, samples were immersed in saline and immediately subjected to biomechanical three-point bending tests using an electronic dynamic and static universal material testing machine (ElectroForce 3200 Series III, Bose, USA). The tibia was placed on two supports with 8 mm apart, and a 0.5 N preload was applied perpendicular to the midpoint of the anterior tibial shaft. The bending load was applied at a rate of 0.167 mm/s until failure. Ultimate load (N), ultimate stiffness (N/mm), and energy to failure (mJ) of tibial midshaft were calculated from the load/displacement curve.

Bone histomorphometry

Another separate set of experiment was performed to evaluate dynamic and static bone histomorphometry. After sacrifice, tibial specimens were cut longitudinally into two pieces along the sagittal plane. One piece was embedded in methylmethacrylate, and then sliced transversely into 50-μm-thick serial sections on a diamond saw microtome cutting system (Leica 2500E; Leica SpA, Milan, Italy). Then, sections were imaged under a fluorescence microscopy (LEICA DM LA; Leica Microsystems, Heidelberg, Germany) to detect and quantify dynamic trabecular bone histomorphometric indices, including mineral apposition rate (MAR, μm/day), mineralizing surface/bone surface (MS/BS, %), and bone formation rate/bone surface (BFR/BS, $\mu m^3/\mu m^2$/day). Another piece was immersed in 4% PFA for 2 days, decalcified in 10% ethylenediamine tetraacetic acid (EDTA) for 4 weeks, embedded in paraffin, and then sliced transversely to 5-μm-thick sections. Sections were stained with hematoxylin-eosin (H&E) to assess osteocyte viability. Tartrate resistant acid phosphatase (TRAP) staining (#387A-1KT, Sigma) and Runx2 immunohistochemical staining (1:200, #sc-390351, Santa Cruz, CA, USA) were performed to evaluate the number of osteoclasts and osteoblasts on trabecular bone surface, respectively. Immunohistochemical staining of sclerostin (1:200, #21933-1-AP, Proteintech, Wuhan, China) and RANKL (1:200, #23408-1-AP, Proteintech) was also performed to determine their expression in osteocytes.

In vitro cell viability and apoptosis assays

The cell viability was determined by the CCK-8 assay. Cells were plated at a concentration of 5×10^3 cells/well in 96-well plates, and then 10 μl CCK-8 solution (#C0037, Beyotime, Shanghai, China) was added into each well to incubate at 37°C for 2 h. The absorbance at 450 nm was measured by a microplate spectrophotometer (Bio-Rad, Philadelphia, PA, USA). The cell apoptosis was measured using an Annexinv-FITC/PI

apoptosis detection kit (KeyGEN, Jiangsu, China). Cells were suspended using 500 μl binding buffer, and then incubated with 5 μl Annexin V-fluorescein isothiocyanate (FITC) and 5 μl propidium iodide (PI) at room temperature for 15 min. The cell apoptosis was then analyzed by a flow cytometry using the 488 nm excitation and 530/630 nm emission wavelengths (CytomicsTM FC 500, Beckman Coulter, CA, USA).

Glucose uptake assays

For the 2-NBDG assays, cells were grown in 6-well plates until reaching 80-90% confluence, and glucose uptake was determined by adding 2-NBDG (Sigma) to the cell culture medium at 100 μM for 1 h. Then, cells were centrifuged at 800 rpm for 5 min and resuspended in PBS supplemented with 2% FBS for flow cytometric analysis. Fluorescence was measured using the 488 nm excitation and 530 nm emission to detect FITC under the flow cytometry. For the 2-DG assays, the cell culture medium was replaced with glucose-free HEPES buffer (pH: 7.4) supplemented with 0.3 μCi/mL [^{3}H]-2-DG. After incubation for 15 min, the scintillating solution was added to the cell lysates, and the radioactivity was measured using a liquid scintillation counter (Tri-Carb 2900TR, PerkinElmer, Waltham, MA, USA).

Assays for metabolic products

An enhanced ATP bioluminescence assay kit (#S0027, Beyotime) was employed to determine the intra- and extracellular ATP levels using cell lysates and culture media according to the manufacturer's instructions, respectively. A commercial ADP (#ab83359, Abcam, Cambridge, UK) assay kit and an AMP (#ab273275, Abcam) assay kit were employed to measure intracellular ADP and AMP concentrations using cell lysates, respectively. To measure the PDH activity in cells, a PDH activity colorimetric assay kit (#K679-100, BioVision, CA, USA) was used according to the manufacturer's instructions. Intracellular glutamate and glutamine concentration were detected using a glutamate assay kit (#ab83389) and a mouse glutamine ELISA assay kit (#ml063302, Enzyme-linked Biotechnology, Shanghai, China), respectively. Intracellular acetyl-CoA and α-KG levels were measured using acetyl-CoA (#ab87546, Abcam) and methyl-α-KG (#ab83431, Abcam) assay kits, respectively. The optical density or luminescence values were measured by microplate reader (Spark, Tecan Trading AG, Switzerland).

Mitochondrial mass, membrane potential, and shape assays

The MLO-Y4 cells were stained with the JC-1 reagent (2 μM, #HY-15534, MedChemExpress, NJ, USA) and MitoTracker Green (0.1 μM, #HY-135056, MedChemExpress) at 37°C for 30 min to assess the mitochondrial membrane potential and mitochondrial mass, respectively. Then, cells were subjected to flow cytometric analysis. To visualize the mitochondrial shape, MLO-Y4 cells were fixed within 3% glutaraldehyde, postfixed with 1% osmium tetroxide followed by 2% uranyl acetate, dehydrated through a series of ethanol gradients, and embedded. Ultrathin sections were isolated on nickel fitters, stained with uranyl acetate followed by lead citrate, and viewed under a transmission electron microscope (HT-7800; Hitachi, Tokyo, Japan).

Osteocytic HIF-1α immunofluorescence imaging

The MLO-Y4 cells were fixed with 4% PFA, permeabilized with 0.1% Triton X-100, and then incubated with the primary antibody against HIF-1α (1:500, #ab179483, Abcam). Then, cells were counterstained with DAPI (#C1002, Beyotime), and the spatial distribution

of HIF-1α in nucleus and cytoplasm was visualized using the Olympus FV3000 confocal microscope.

Osteoblastic ALP staining/activity assays and alizarin red staining

Primary osteoblasts were fixed within 4% PFA and incubated in BCIP/NBT solution (33 μl BCIP, 66 μl NBT, and 10 ml Chromogenic buffer) for 1 h from an ALP color development kit (Beyotime, Shanghai, China). Images of ALP positive cells were captured under an optical microscope (Leica DM LA, Leica Microsystems, Wetzlar, Germany). The ALP activity was quantified using an ALP activity assay kit (Nanjing Jiancheng Bioengineering Institute, Nanjing China) based on protein quantification of proteins isolated with RIPA buffer was performed using a BCA assay kit (Beyotime). For osteoblast mineralization assay, cells were fixed within 4% PFA and stained with 2% Alizarin Red S solution (pH=4.2; Cyagen, Jiangsu, China) at 37°C for 30 min. Mineralized matrix formation with a bright red-colored deposition was captured under the optical microscope. Then, cells were dissolved with 10% acetic acid for 30 min, and the extracted stain was quantified for the absorbance at 590 nm.

Osteoclast TRAP staining and cytoskeleton staining

For TRAP staining, RAW264.7 cells were fixed in 4% PFA, and then stained using a commercial TRAP staining kit (Sigma) in accordance with the manufacturer’s instructions. TRAP-positive multinucleated cells containing three or more nuclei were identified as osteoclasts. For osteoclastic cytoskeleton staining, cells were fixed in 4% PFA, permeabilized with 0.1% Triton X-100, and then stained with 50 μl/ml FITC-conjugated phalloidin (#P5282, Sigma) for 40 min followed by DAPI staining for 10 min. Then, fluorescence images were captured under the confocal microscope.

RNA isolation and quantitative real-time PCR analysis

Total RNA was collected using an RNA isolation kit (#TR205, Beijing Tianmo Biotechnology, China) according to the manufacturer's instructions. After determination of the RNA concentration and quality based on ABI QuantStudio, complementary DNA (cDNA) was prepared with 1000 ng total RNA using a cDNA synthesis kit (#RR037A, TaKaRa, Japan). qPCR was performed on QuantStudio 7 Flex real-time PCR detection system (Thermo Fisher Scientific Inc., MA, USA) with SYBR green master mix, and the PCR primer sequences are listed in Table S2. β-actin was used as internal reference control to normalize the mRNA levels. Data were analyzed by the $2^{-\Delta\Delta Ct}$ method.

Western blotting

Total proteins were isolated in RIPA lysis buffer supplemented with protease and phosphatase inhibitors for 30 min on ice, and the protein concentration was measured using a BCA protein assay kit (#P0012, Beyotime). Then, protein samples (20 μg) were separated by 10% polyacrylamide gel electrophoresis (SDS-PAGE), transferred to polyvinylidene fluoride (PVDF) membrane (0.45 μm) on ice, and then blocked with 5% skim milk for 1 h. The membranes were incubated overnight at 4°C with primary antibodies against Runx2 (1:1000, sc-390351, Santa Cruz), RANKL (1:1000, #23408-1-AP, Proteintech), HIF-1α (1:1000, #ab179483, Abcam), PDK1 (1:1000, #18262-1-AP, Proteintech), GLS2 (1:1000, #ab113509, Abcam), β-catenin (1:1000, #51067-2-AP, Proteintech), OPG (1:1000, #sc-390518, Santa Cruz), DKK1 (1:2000, #21112-1-AP, Proteintech), Col1a1 (1:1000, #ab260043, Abcam), Osx (1:1000, #bs-1110R, Bioss, Beijing, China), NFATc1 (1:5000, #66963-1-Ig, Proteintech), Calcr (1:300, #20868-1-AP, Proteintech), Ctsk (1:1000, #sc-48353, Santa Cruz), TRAP

(1:2000, #ab52750, Abcam), β-actin (1:4000, #20536-1-AP, Proteintech), Lamin B1 (1:4000, #12987-1-AP, Proteintech) and then transferred to the HRP-conjugated secondary antibody (1: 5000, #ab6789/ab6721, Abcam) or fluorescent-labeled secondary antibody (1: 1000, #ab150077, Abcam) for 1 h (details shown in Table S3). The visualization was performed using the chemiluminescence reagent, and the grey values of the target protein bands were analyzed using the ImageJ software.

Transcription factor prediction and luciferase reporter gene assays

The marked-up genomic sequence of 3000 bps of *GLS2* was downloaded from the Ensembl database. The potential binding sites of the top 3000 bases and transcription factors filtered by GO analysis were predicted on http://bioinfo.life.hust.edu.cn/AnimalTFDB/#!/tfbs_predict, and the base sequences with $P<10^{-5}$ were obtained. The transcription factors potentially recognized the transcription initiation site of *GLS2* were determined by combining the transcription factor prediction with GO analysis. The identified *GLS2* transcription initiation site was then truncated at -2800, -900 and -700 binding site, and transfected into cells with the PGL3-basic vector. The transcription factor HIF-1α sequences binding to *GLS2* promoter in the predicted sequences were determined by detecting the relative luciferase activity of the three truncated sequences (PI, PII and PIII) of HIF-1α via a commercial luciferase assay kit (Beyotime). Data were normalized against the Renilla luciferase activity. The PGL3-basic plasmid was used as a negative control.

ChIP assays

ChIP was conducted using a commercial EZ-ChIP™ kit (#17-371, Millipore, Billerica, MA, USA) following the manufacturer's instructions. Cells were fixed with formaldehyde and incubated with glycine, and the chromatin was then sheared by sonication. The antibody anti-HIF-1α (#ab179483, Abcam) and normal IgG (#ab172730, Abcam) were used to precipitate the crosslinked protein-DNA complexes. Then, qRT-PCR was performed to analyze the precipitated DNA. The GLS2 primer sequences are shown in Table S2. Primers which amplify regions lacking HRE of PDK1 were used as a negative control. The percentage of total chromatin input was quantified. Input was employed as the DNA after dispergation and purification.

RNA interference

A total of three different HIF-1α shRNAs with non-overlapping sequences (as listed in Table S4) were designed and verified by GenePharma (Shanghai, China). These shRNAs were individually packed into the lentiviral LV-3 (pGLVH1/GFP+Puro) vector. The lentiviral shRNA (20 μl/ml) were added to cells for 24 h. Transfected cells were selected in a medium containing 2 μg/ml puromycin for 2 weeks to remove non-transfected cells. The non-sense scramble control lentiviral shRNA was employed as the negative control. The efficiency of HIF-1α silencing was evaluated using western blotting assays.

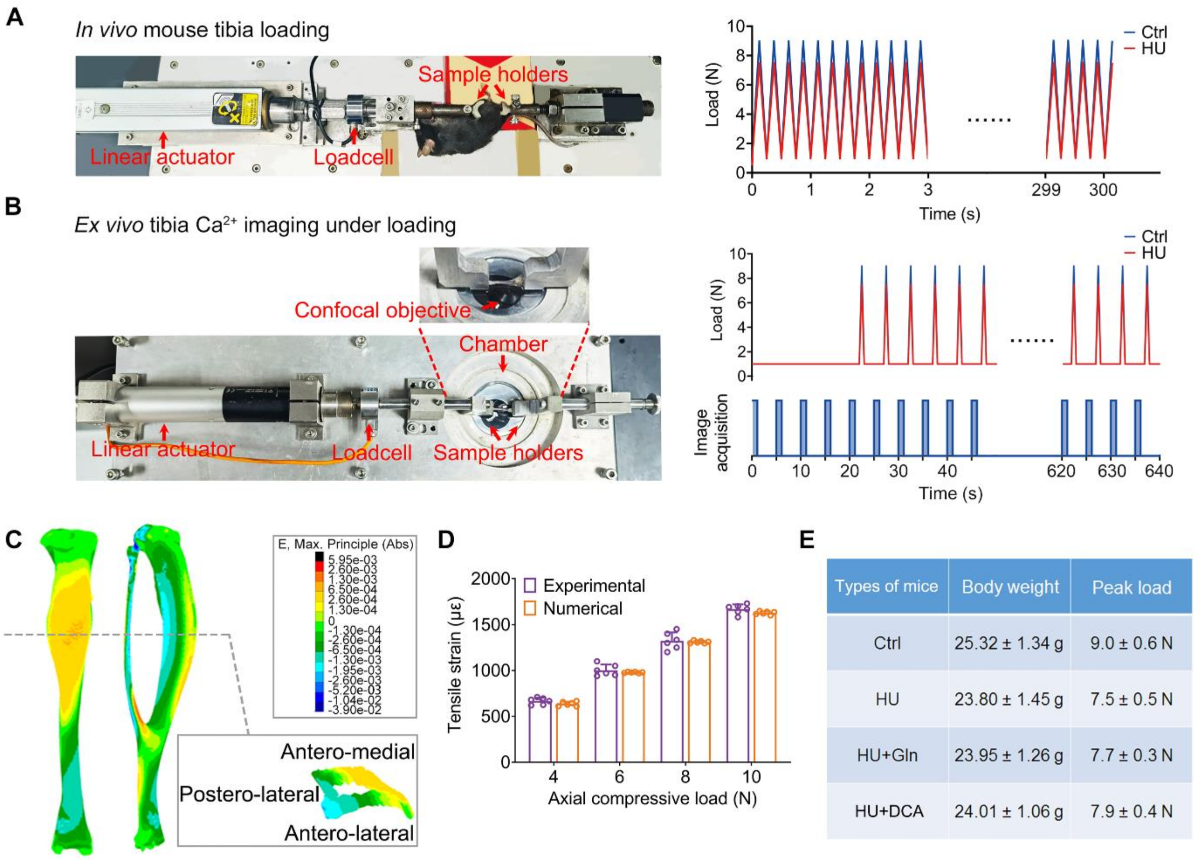

Types of mice	Body weight	Peak load
Ctrl	25.32 ± 1.34 g	9.0 ± 0.6 N
HU	23.80 ± 1.45 g	7.5 ± 0.5 N
HU+Gln	23.95 ± 1.26 g	7.7 ± 0.3 N
HU+DCA	24.01 ± 1.06 g	7.9 ± 0.4 N

Figure S1, related to Figure 1 and Figure 2. The mechanical loading setup and characterization of the strain distribution under compressive loading. **(A)** The custom-designed mechanical loading device generating uniaxial compressive loading to the mouse tibia *in vivo*, which consisted of the linear actuator, linear guide, sample holders (including an immovable holder and a movable holder), and loadcell. A waveform of cyclic ramp loading was applied on the mouse tibia. **(B)** The custom-designed mechanical loading device generating uniaxial compression to the intact mouse tibia *ex vivo* for visualizing Ca^{2+} fluorescence in bone cells *in situ*. After incubation with the Ca^{2+} indicator Calbryte-520 AM, the mouse tibia was transferred to the chamber of the mechanical loading device that was filled with the tissue culture medium to maintain the bone cell viability. A novel synchronized loading/imaging technique was employed to avoid the drift of confocal objective focus during dynamic loading. A dwell time of 4 s was applied between each cycle. Fluorescence images were captured at 1.1 s/frame during the dwell time after each loading cycle. **(C)** MicroCT-based finite element analysis for the three-dimensional strain distributions when the axial peak load of 9 N was applied on the mouse tibia. **(D)** Comparison of strain measurements between MicroCT-based finite element analysis and strain gauges. *n*=6/group. **(E)** The peak loads corresponding to identical strain (1500 με) on the tibial surfaces in control and hindlimb unloading mice. Graphs represent mean ± SD. All analyses were performed by two-tailed unpaired Student's t test.

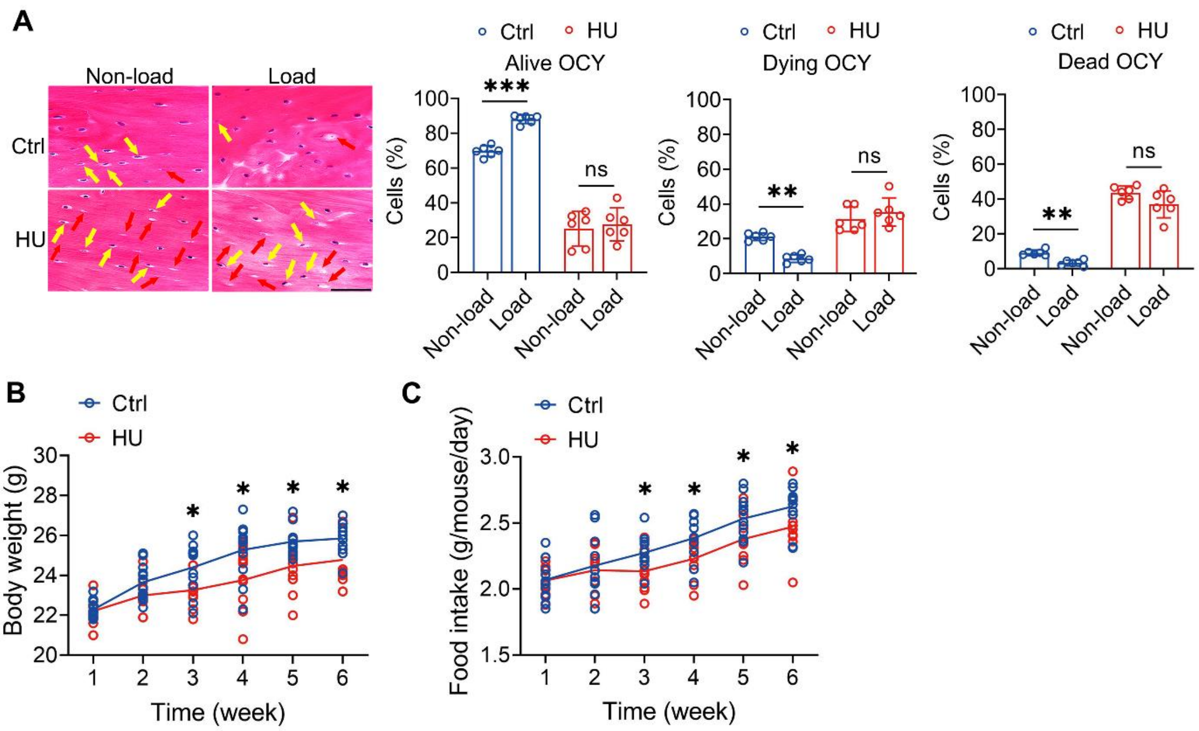

Figure S2, related to Figure 1. Comparison of the body weight, food intake and osteocyte viability in experimental animals. **(A)** The effects of mechanical loading on osteocyte viability in mice exposed to previous hindlimb unloading via H&E staining. *n*=6/group. **(B and C)** Comparison of the body weight and food intake in normal mice and mice with hindlimb unloading. *n*=12/group. Graphs represent mean ± SD. **A** ***P*<0.01 and ****P*<0.001 by 2-way ANOVA with Bonferroni's post test. **B, C** **P*<0.05 by two-tailed unpaired Student's t test. Scale bar: **A** 50 μm.

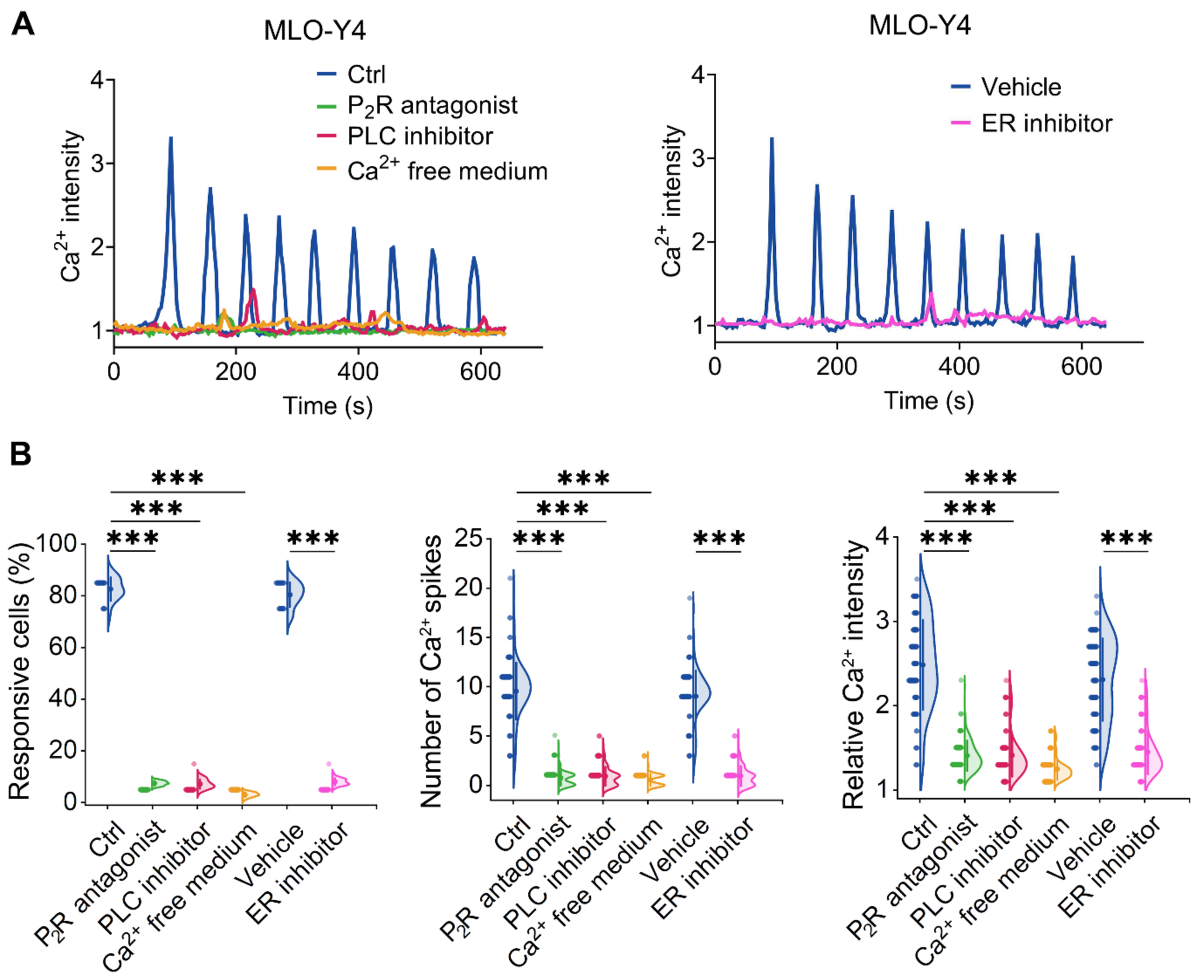

Figure S3, related to Figure 2. Effects of the P_2 receptor (P_2R) antagonist (PPADS), phospholipase C (PLC) antagonist, depletion of the endoplasmic reticulum Ca^{2+} stores, and Ca^{2+} free culture medium on intracellular Ca^{2+} dynamics in SMG-exposed osteocytic MLO-Y4 cells *in vitro* under subsequent steady fluid flow stimulation. **(A)** Representative intracellular Ca^{2+} signaling curves in SMG-exposed MLO-Y4 cells treated with the P_2R antagonist PPADS (150 μM), PLC antagonist neomycin (1 mM), Ca^{2+} free culture medium, thapsigargin (1 μM; depletion of the endoplasmic reticulum Ca^{2+} stores), and DMSO (vehicle control) in SMG-exposed MLO-Y4 cells under subsequent fluid flow stimulation. **(B)** The corresponding statistical comparison of intracellular Ca^{2+} signaling. n=90/group. Graphs represent mean ± SD. **B** ***P<0.001 by 1-way ANOVA with Bonferroni's post test.

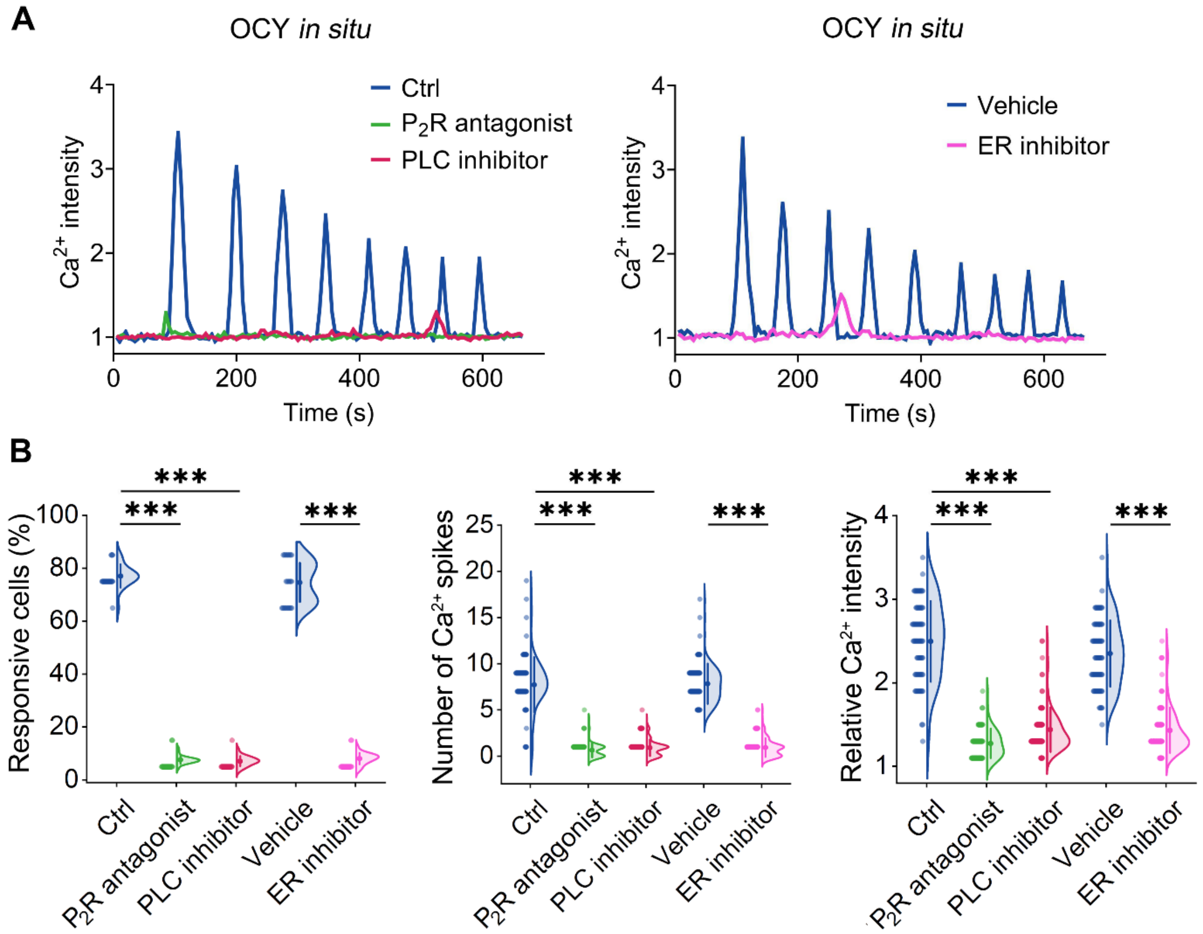

Figure S4, related to Figure 2. Effects of the P_2 receptor (P_2R) antagonist (PPADS), phospholipase C (PLC) antagonist and depletion of the endoplasmic reticulum Ca^{2+} stores on intracellular Ca^{2+} dynamics of osteocytes *in situ* in tail-suspended mice in response to tibial mechanical reloading. **(A)** Comparison of real-time intracellular Ca^{2+} signaling of tibial osteocytes *in situ* in tail-suspended mice treated with the P_2R antagonist PPADS (150 μM), PLC antagonist neomycin (1 mM), thapsigargin (1 μM; depletion of the endoplasmic reticulum Ca^{2+} stores), and DMSO (vehicle control) under cyclic compressive loading. **(B)** The corresponding statistical comparison of intracellular Ca^{2+} signaling (*n*=72 cells for Ctrl, *n*=70 cells for P_2R antagonist, *n*=95 cells for PLC inhibitor, *n*=72 cells for Vehicle, and *n*=95 cells for ER inhibitor). Graphs represent mean ± SD. **B** ****P*<0.001 by 1-way ANOVA with Bonferroni's post test.

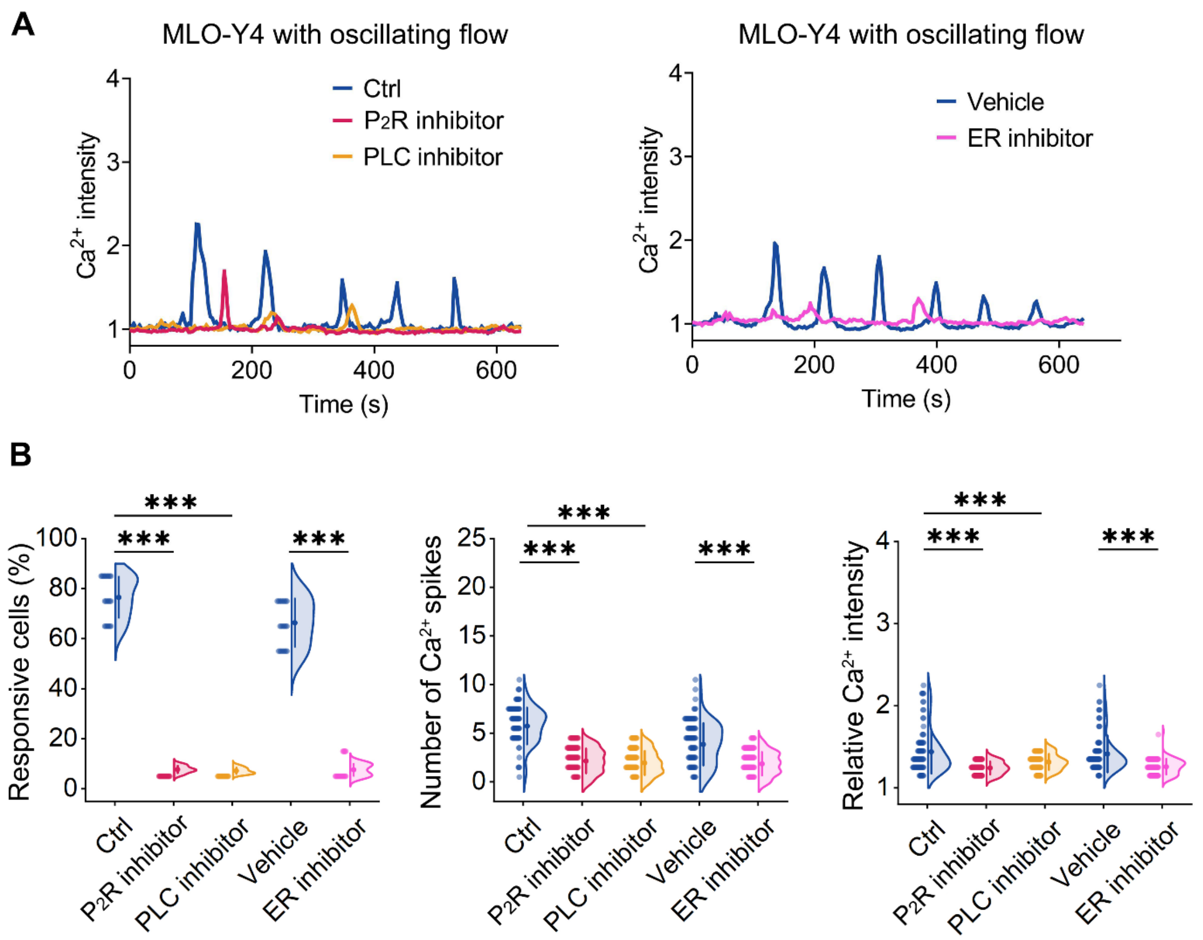

Figure S5, related to Figure 2. Effects of oscillating fluid flow (1 Hz) on intracellular Ca^{2+} dynamics in SMG-exposed osteocytic MLO-Y4 cells and their ATP signaling-dependent mechanism. **(A)** Comparison of intracellular Ca^{2+} signaling in SMG-exposed MLO-Y4 cells treated with P_2 receptor (P_2R) antagonist PPADS (150 μM), PLC antagonist neomycin (1 mM), thapsigargin (1 μM; depletion of the endoplasmic reticulum Ca^{2+} stores), and DMSO (vehicle control) in SMG-exposed MLO-Y4 cells under subsequent oscillating fluid flow stimulation. **(B)** The corresponding statistical comparison of intracellular Ca^{2+} signaling. *n*=90/group. Graphs represent mean ± SD. **B** $***P<0.001$ by 1-way ANOVA with Bonferroni's post test.

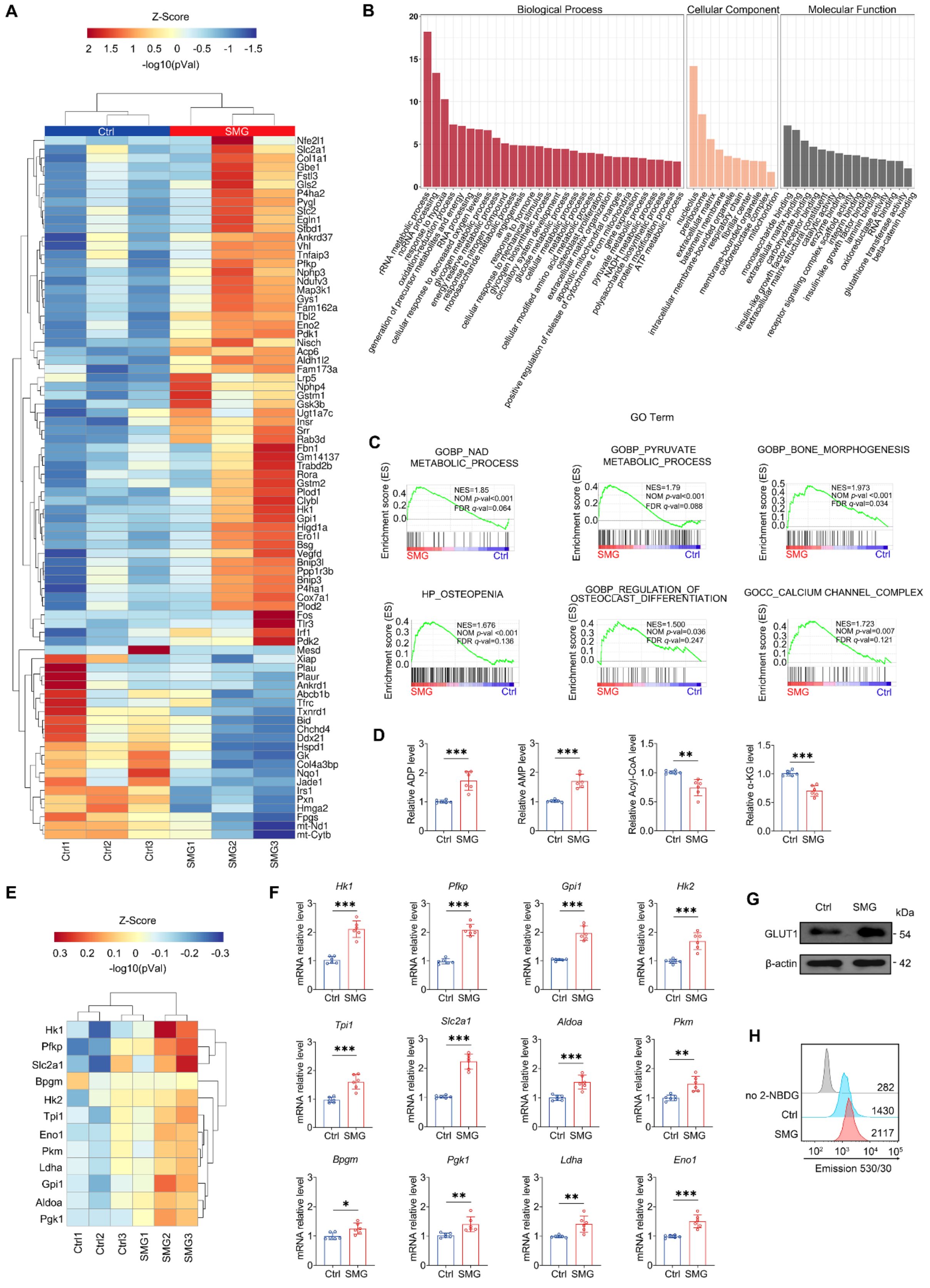

Figure S6, related to Figure 3. The RNA sequencing, analysis of glycolysis-related gene expression, and glucose uptake assays in MLO-Y4 osteocytic cells exposed to SMG for 48 h.

(A) The heatmap showing the most significantly altered genes (top 80) related to cell metabolism. *n*=3/group. **(B)** The GO analysis for Biological Process, Molecular Function, and Cellular Component of up- and down-regulated genes. *n*=3/group. **(C)** Gene set enrichment analysis (GSEA) showing the significantly enriched pathways, including NAD and pyruvate metabolic processes, bone morphogenesis, osteopenia, osteoclast differentiation, and calcium channel complex. *n*=3/group. **(D)** Assays for intracellular ADP, AMP, acetyl-CoA, and α-ketoglutarate concentrations. *n*=6/group. **(E)** The heatmap showing the alteration of genes associated with glucose transporting and glycolysis-related pathways. *n*=3/group. **(F)** qRT-PCR assays confirming the RNA sequencing results for the expression of glucose transporting and glycolysis-related genes. *n*=6/group. **(G)** Western blotting assays for the protein expression of glucose transporter 1 (GLUT1). **(H)** 2-NBDG assays for glucose uptake. *n*=3/group. Graphs represent mean ± SD. **D, F** **P*<0.05, ***P*<0.01, and ****P*<0.001 by two-tailed unpaired Student's t test.

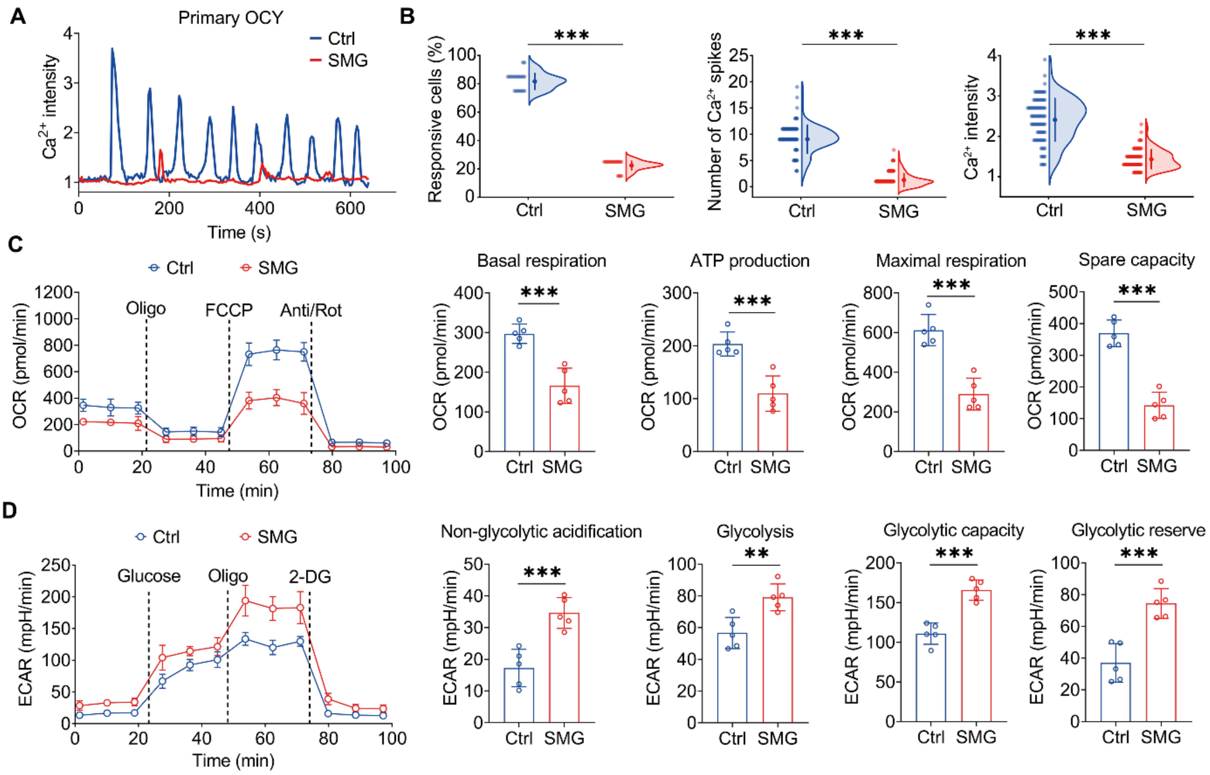

Figure S7, related to Figure 2 and Figure 3. Effects of SMG exposure on intracellular Ca^{2+} dynamics in response to fluid flow stimulation and energy metabolism in primary osteocytes. Primary osteocytes were isolated from mouse hindlimbs, and then subjected to SMG exposure for 48 in a rotating bioreactor. **(A and B)** Comparison of intracellular Ca^{2+} oscillatory dynamics in SMG-exposed and normal primary osteocytes in response to steady fluid flow stimulation (n=90 cells for Ctrl and n=85 cells for SMG). **(C and D)** The high-throughput Seahorse assays to simultaneously monitor cellular oxygen consumption rate (OCR) and extracellular acidification rate (ECAR) in living normal and SMG-exposed primary osteocytes. n=5/group. Graphs represent mean ± SD. **P<0.01 and ***P<0.001 by two-tailed unpaired Student's t test.

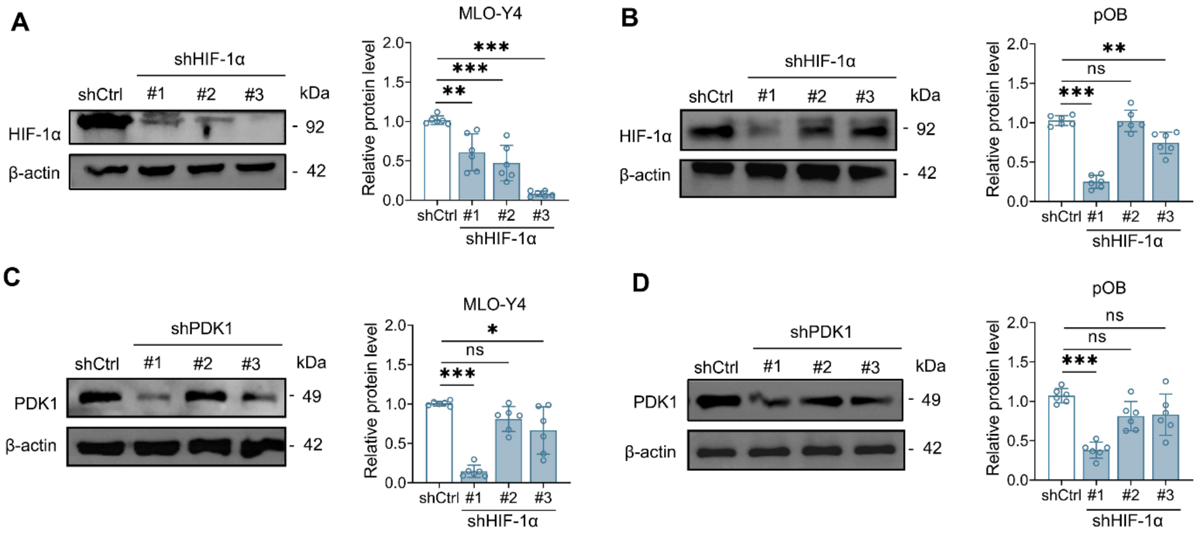

Figure S8, related to Figure 4. Western blotting assays testing the gene knockdown efficiency of HIF-1α and PDK1 in osteocytic MLO-Y4 cells and primary osteoblasts after lentiviral transduction by different shRNAs. *n*=6/group. Graphs represent mean ± SD. **P*<0.05, ***P*<0.01, and ****P*<0.001 by 1-way ANOVA with Bonferroni's post test.

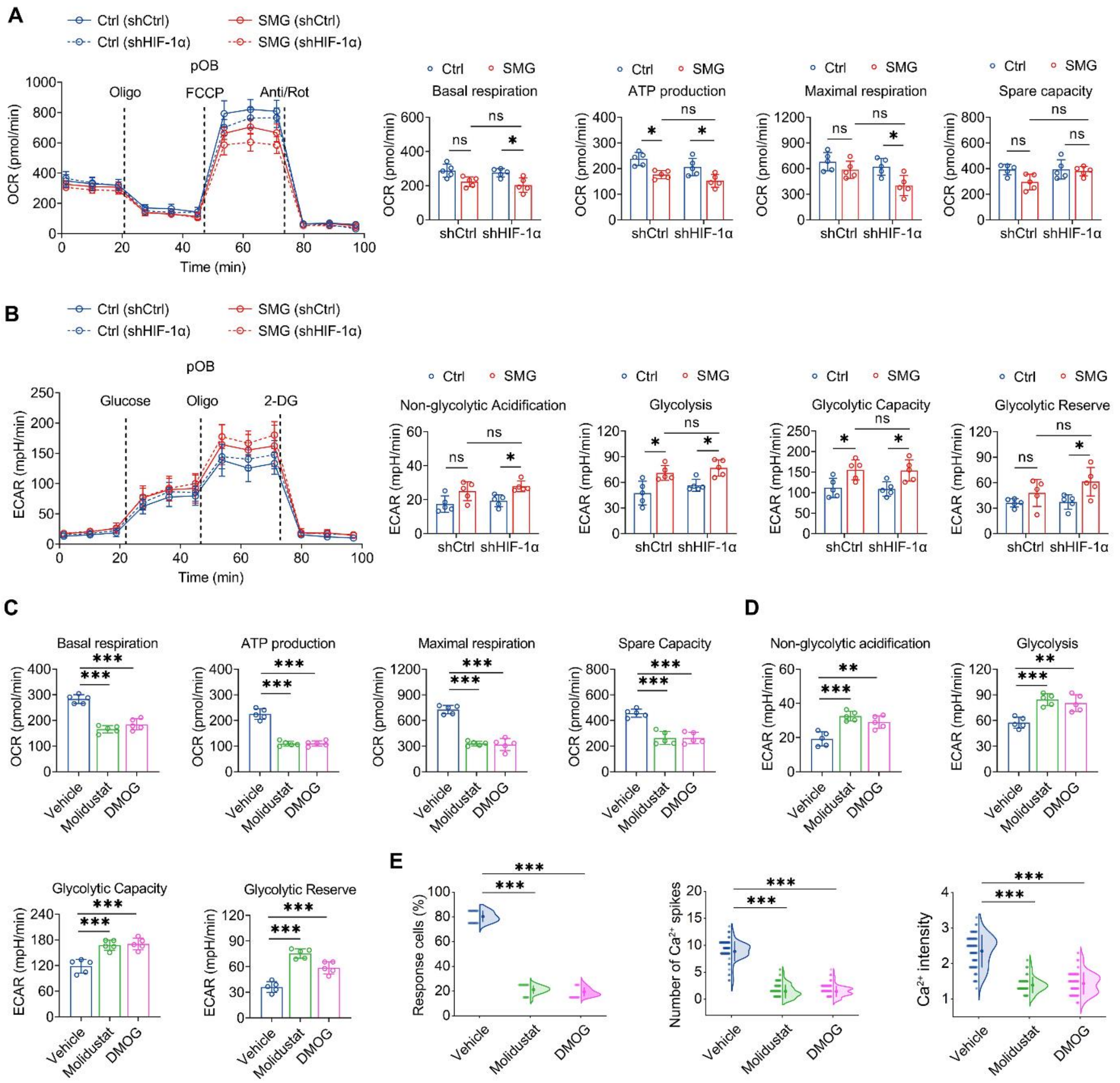

Figure S9, related to Figure 4. Effects of HIF-1α silencing on energy metabolism in SMG-exposed primary osteoblasts, and HIF-1α activation on energy metabolism and mechanoresponse in osteocytic MLO-Y4 cells. **(A)** The Seahorse assay to monitor cellular oxygen consumption rate (OCR) in normal and SMG-exposed primary osteoblasts infected with shCtrl and shHIF-1α lentivirus. *n*=5/group. **(B)** The Seahorse assay to monitor extracellular acidification rate (ECAR) in normal and SMG-exposed primary osteoblasts infected with shCtrl and shHIF-1α lentivirus. *n*=5/group. **(C and D)** Effects of pharmacologic activation of HIF-1α using molidustat (10 μM) or DMOG (1 mM) on cellular OCR and ECAR in MLO-Y4 cells. *n*=5/group. **(E)** Effects of the molidustat or DMOG treatment on intracellular Ca^{2+} dynamics in MLO-Y4 cells under fluid flow stimulation. *n*=85/group. Graphs represent mean ± SD. **A, B** **P*<0.05 by 2-way ANOVA with Bonferroni's post test. **C~E** ***P*<0.01 and ****P*<0.001 by 1-way ANOVA with Bonferroni's post test.

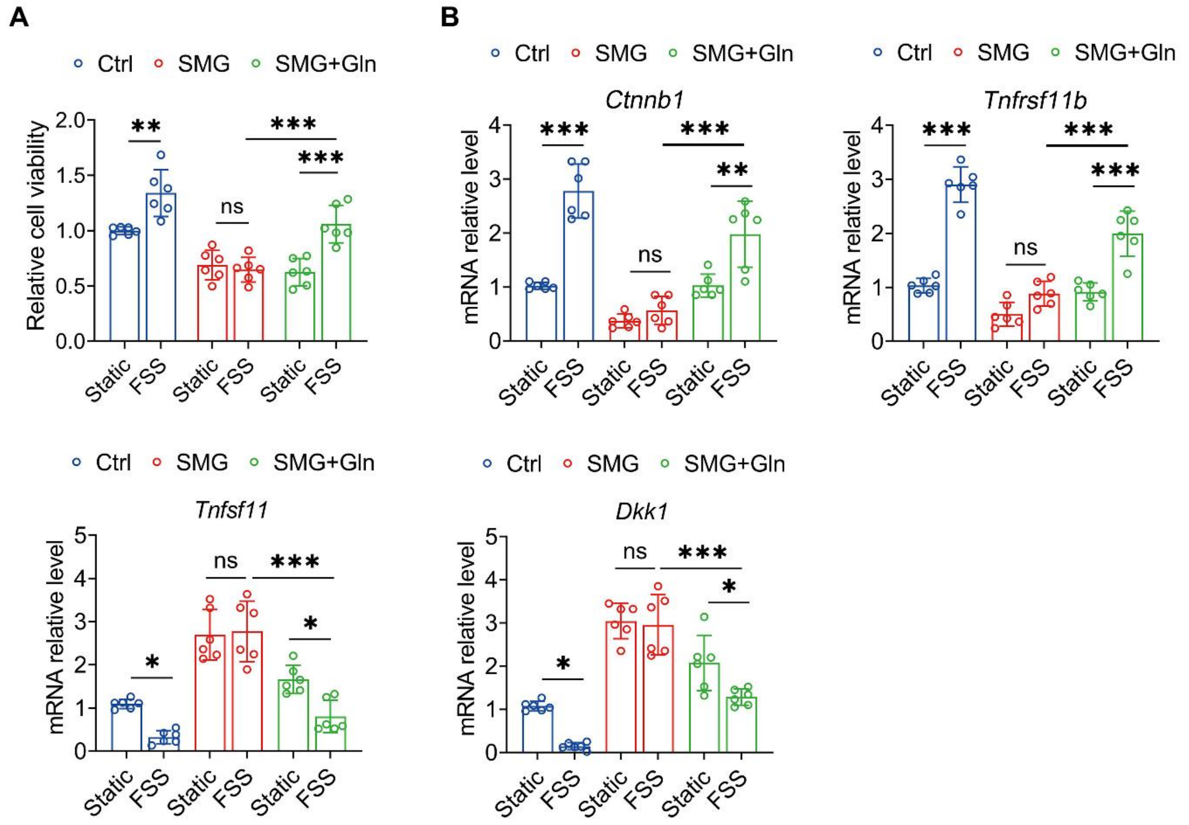

Figure S10, related to Figure 6. Effects of glutamine supplementation on cellular viability and function in response to fluid flow stimulation in MLO-Y4 osteocytic cells exposed to SMG. **(A)** CCK-8-based cell viability assays in normal and SMG-exposed MLO-Y4 cells treated with glutamine. *n*=6/group. **(B)** The gene expression assays in normal and SMG-exposed MLO-Y4 cells treated with glutamine, including *Ctnnb1*, *Tnfrsf11b*, *Tnfsf11*, and *Dkk1*. *n*=6/group. Graphs represent mean ± SD. **P*<0.05, ***P*<0.01, and ****P*<0.001 by 3-way ANOVA with Bonferroni's post test.

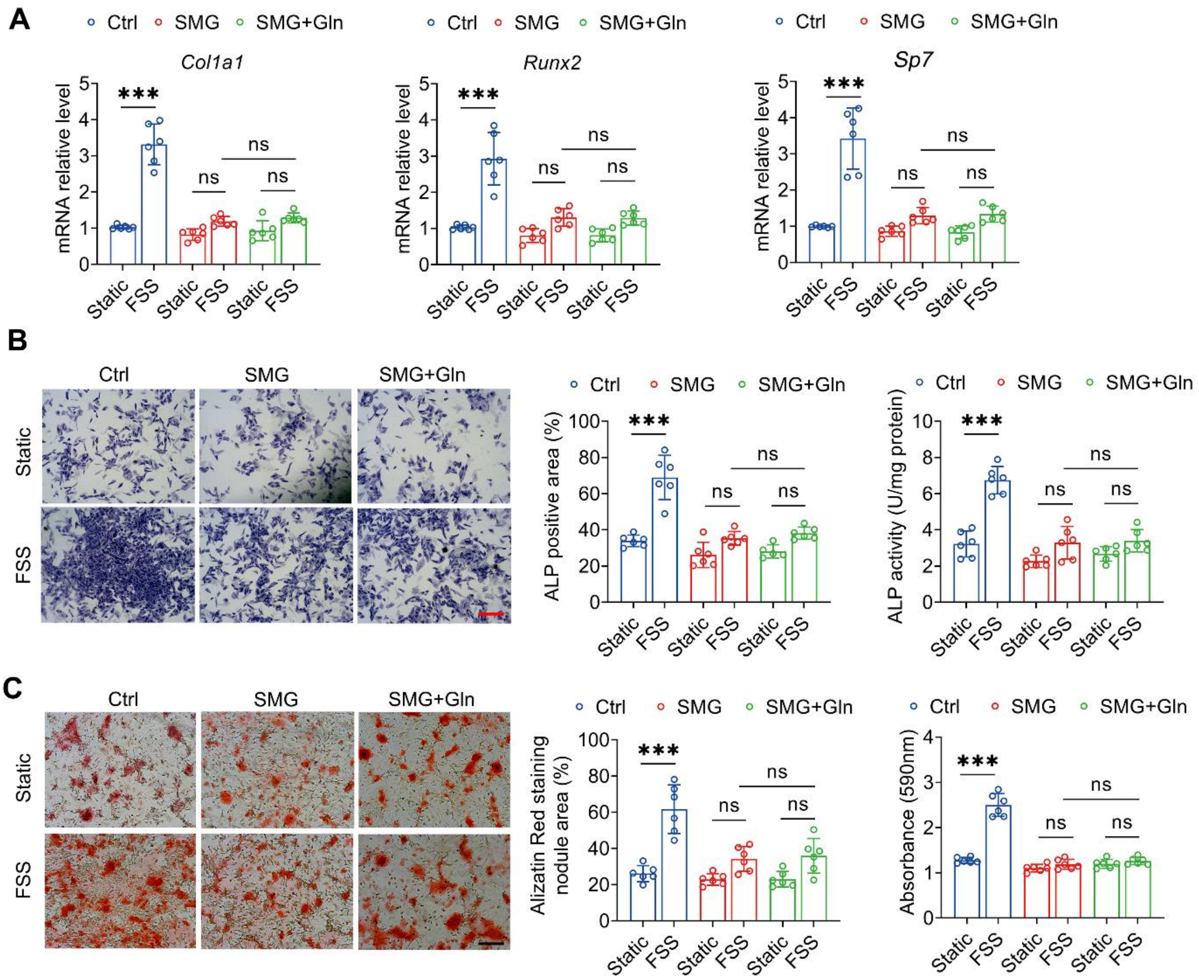

Figure S11, related to Figure 6. Effects of glutamine supplementation on cellular differentiation in response to fluid flow stimulation in primary osteoblasts exposed to SMG. **(A)** qRT-PCR analyses of the gene expression of *Col1a1*, *Sp7*, and *Runx2* following osteogenic medium incubation. *n*=6/group. **(B)** The ALP staining following osteogenic medium incubation. *n*=6/group. **(C)** Alizarin red staining following osteogenic medium incubation. *n*=6/group. Graphs represent mean ± SD. ****P*<0.001 by 3-way ANOVA with Bonferroni's post test. Scale bars: **B, C** 50 μm.

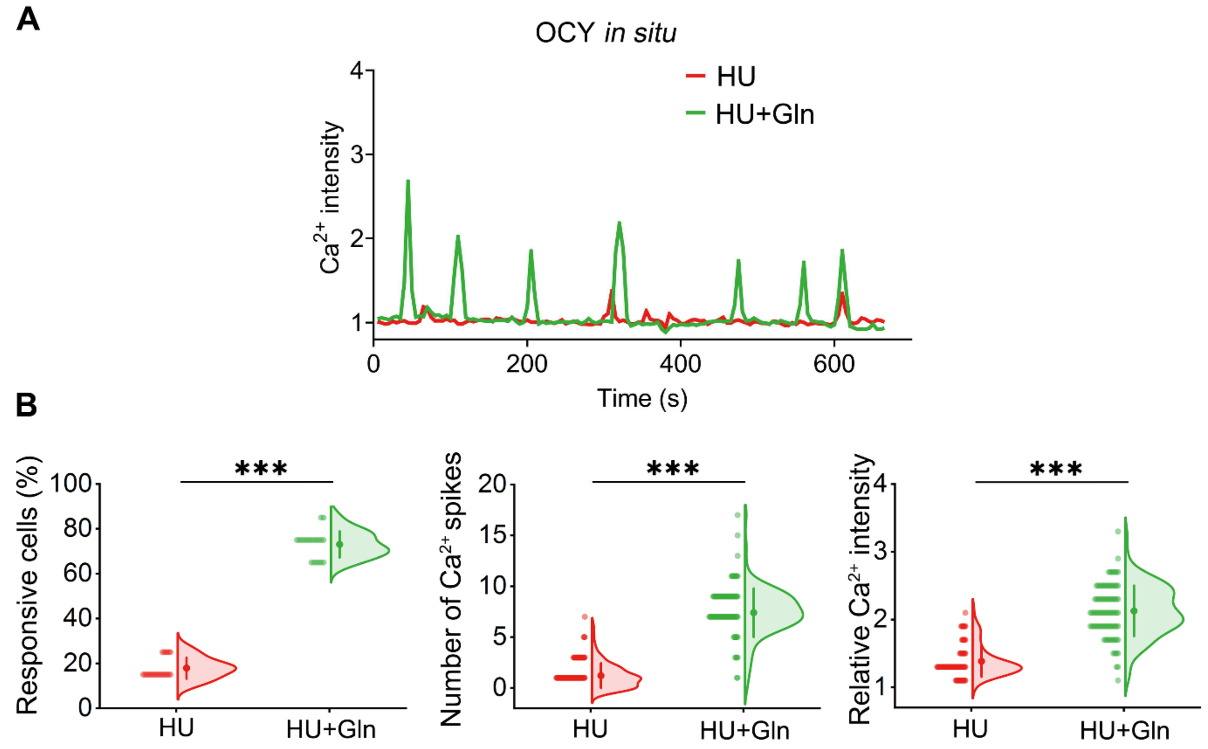

Figure S12, related to Figure 6. Effects of glutamine supplementation on intracellular Ca^{2+} dynamics of osteocytes *in situ* in tail-suspended mice in response to tibial mechanical reloading (*n*=80 cells for HU and *n*=95 cells for HU+Gln). Graphs represent mean ± SD. **B** ****P*<0.001 by two-tailed unpaired Student's t test.

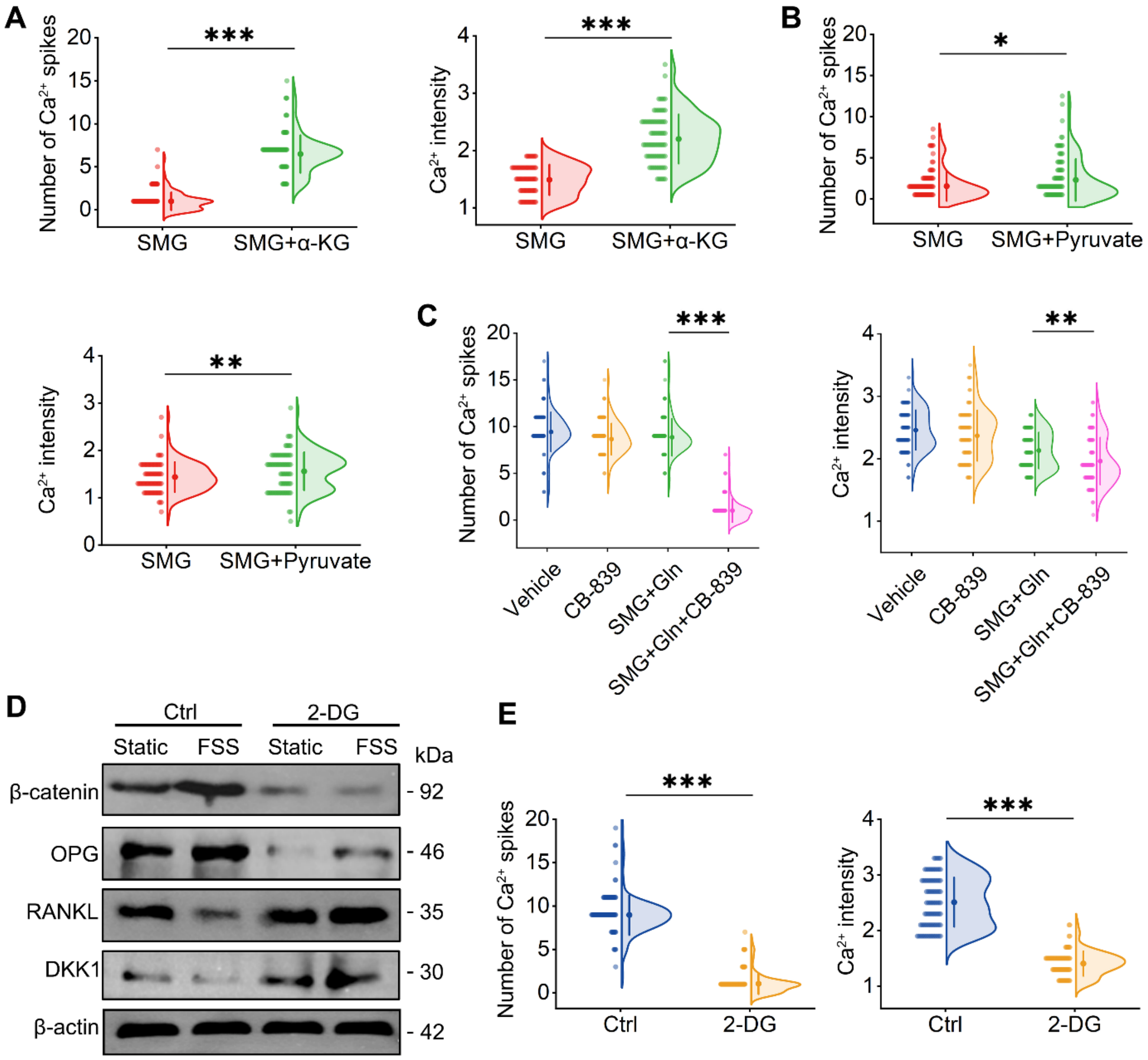

Figure S13, related to Figure 6. Effects of treatment with α-ketoglutarate (α-KG) and pyruvate on mechanoresponse to fluid flow stimulation in MLO-Y4 osteocytic cells exposed to SMG, and effects of blocking glutamine and pyruvate metabolism on mechanoresponse to fluid flow stimulation in normal MLO-Y4 cells. **(A)** Statistical analyses of α-KG treatment on Ca^{2+} signaling parameters in response to fluid flow stimulation in SMG-exposed MLO-Y4 cells. *n*=85/group. **(B)** Statistical analyses of pyruvate treatment on Ca^{2+} signaling parameters in response to fluid flow stimulation in SMG-exposed MLO-Y4 cells. *n*=86/group. **(C)** Statistical analyses of the glutaminase inhibitor CB-839 on Ca^{2+} signaling parameters in response to fluid flow stimulation in normal MLO-Y4 cells and SMG-exposed MLO-Y4 cells supplemented with glutamine. *n*=90/group. **(D and E)** Treatment with 2-DG (blocking the conversion of glucose to pyruvate) on osteocyte-related protein expression and Ca^{2+} signaling parameters in response to fluid flow stimulation in MLO-Y4 cells. *n*=90/group. Graphs represent mean ± SD. **A, B, E** **P*<0.05, ***P*<0.01, and ****P*<0.001 by two-tailed unpaired Student's t test. **C** ***P*<0.01 and ****P*<0.001 by 1-way ANOVA with Bonferroni's post test.

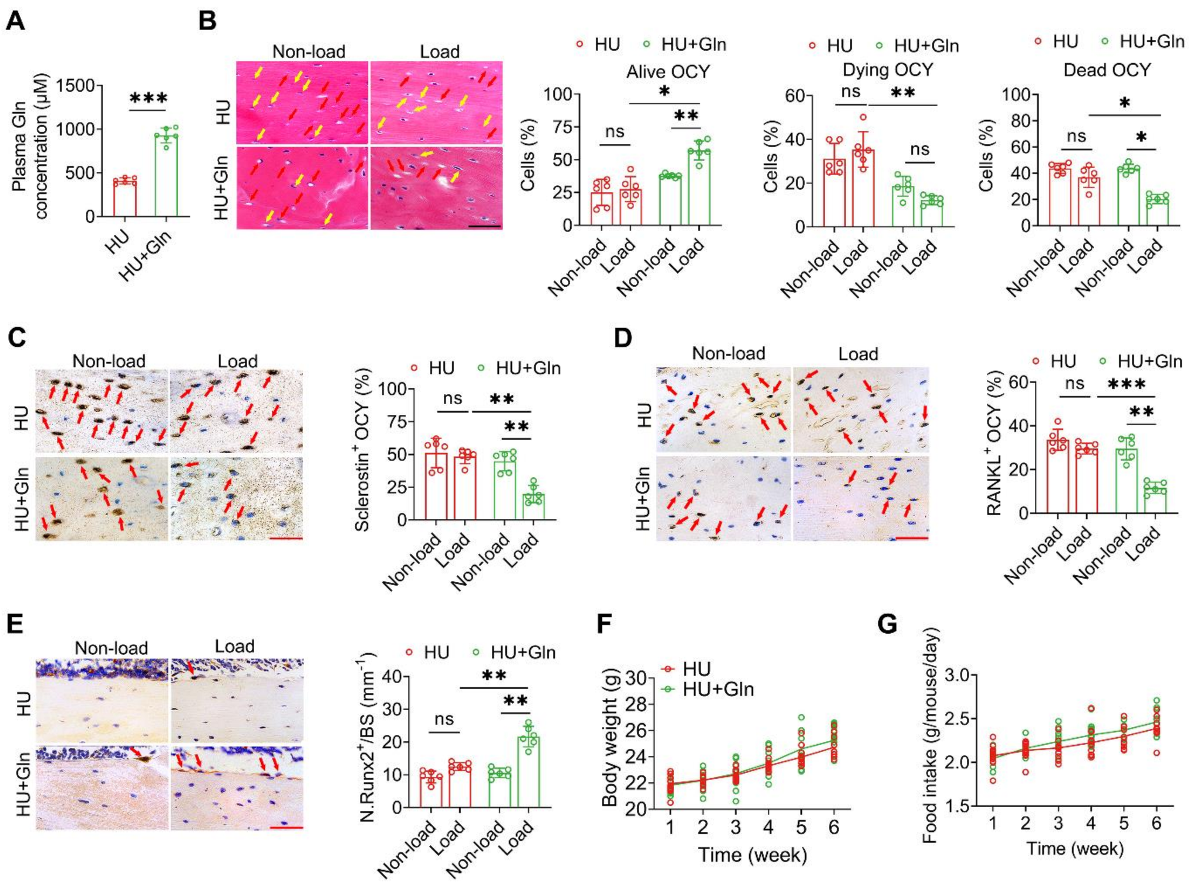

Figure S14, related to Figure 7. Effects of glutamine supplementation on osteocyte viability and function, osteoblast number on bone surface, body weight, and food intake and in tail-suspended mice in response to tibial mechanical reloading. **(A)** Measurements of the circulating glutamine levels using a commercial assay kit in blood samples collected 1 h later at the last day of glutamine injection. *n*=6/group. **(B)** H&E staining assays for osteocyte viability. *n*=6/group. **(C and D)** Immunohistochemical staining assays for osteocytic RANKL and sclerostin expression. *n*=6/group. **(E)** Runx2 immunohistochemical staining labeling osteoblasts on bone surface. *n*=6/group. **(F and G)** Comparison of the body weight and food intake in normal mice, tail-suspended mice, and tail-suspended mice treated with glutamine. *n*=12/group. Graphs represent mean ± SD. **A, F, G** ***P<0.001 by two-tailed unpaired Student's t test. **B~E** *P<0.05, **P<0.01, and ***P<0.001 by 2-way ANOVA with Bonferroni's post test. Scale bars: **B~E** 50 μm.

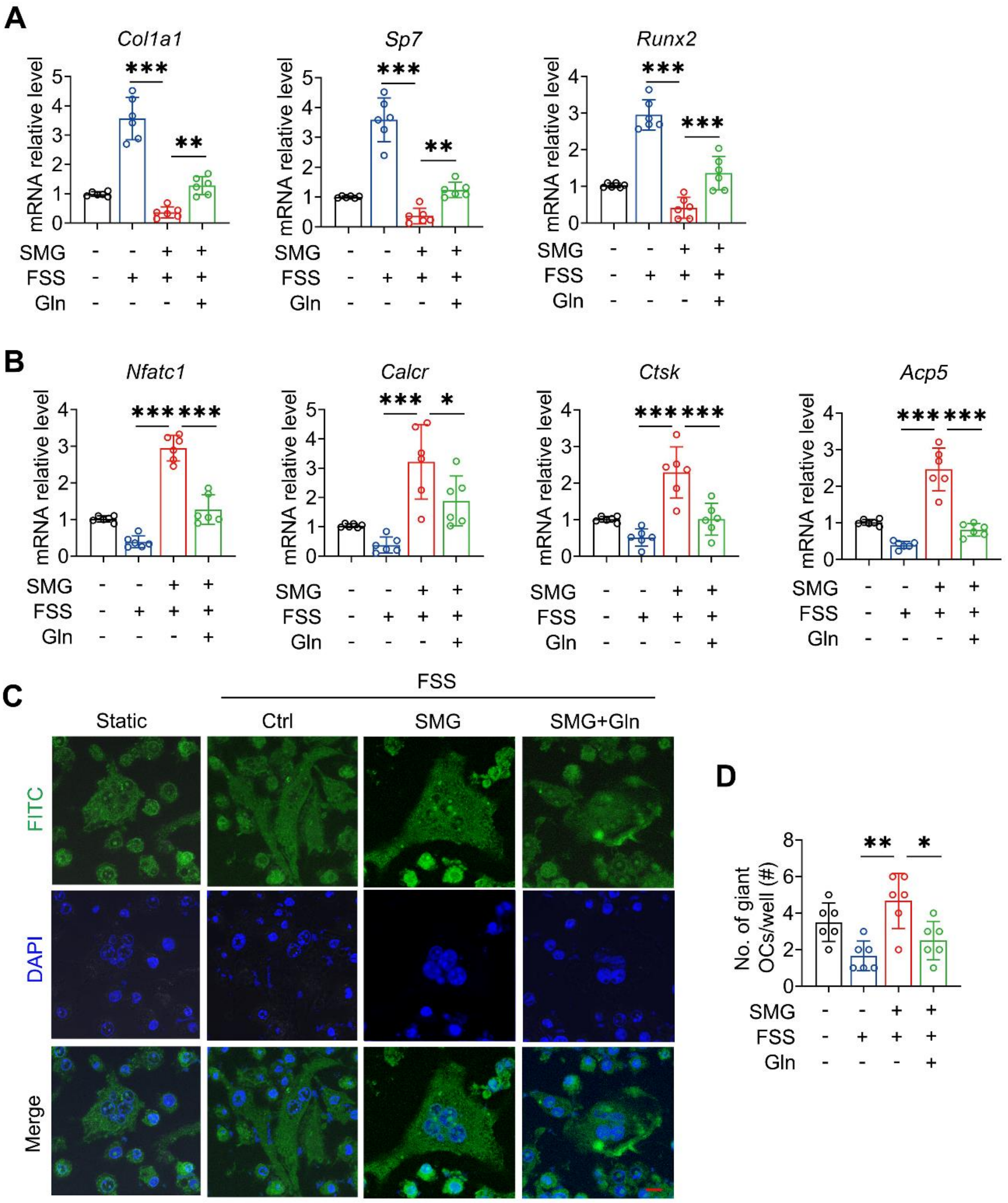

Figure S15, related to Figure 7. Effects of the conditioned medium collected from SMG-exposed osteocytes treated with glutamine in response to fluid flow reloading on the viability and function of SMG-exposed osteoblasts and osteoclasts. **(A)** qRT-PCR analyses of the gene expression associated with osteoblast differentiation, including *Col1a1*, *Sp7*, and *Runx2*. *n*=6/group. **(B)** qRT-PCR analyses of the expression of osteoclast marker genes, including *Nfatc1*, *Calcr*, *Ctsk*, and *Acp5*. *n*=6/group. **(C and D)** F-actin cytoskeleton immunofluorescence staining for osteoclasts using FITC-conjugated phalloidin, and the corresponding quantitative data. *n*=6/group. Graphs represent mean ± SD. **P*<0.05, ***P*<0.01, and ****P*<0.001 by 2-way ANOVA with Bonferroni's post test. Scale bar: **C** 10 μm.

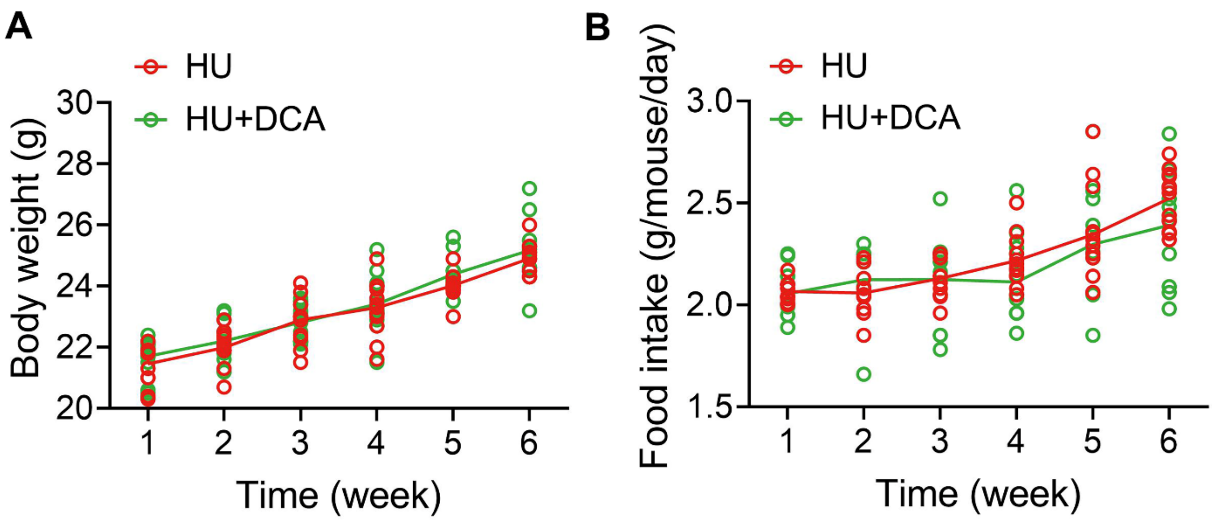

Figure S16, related to Figure 8. Effects of PDK1 antagonist treatment on the body weight and food intake in tail-suspended mice in response to tibial mechanical reloading. *n*=12/group. Graphs represent mean ± SD. Statistical analyses were performed by two-tailed unpaired Student's t test.

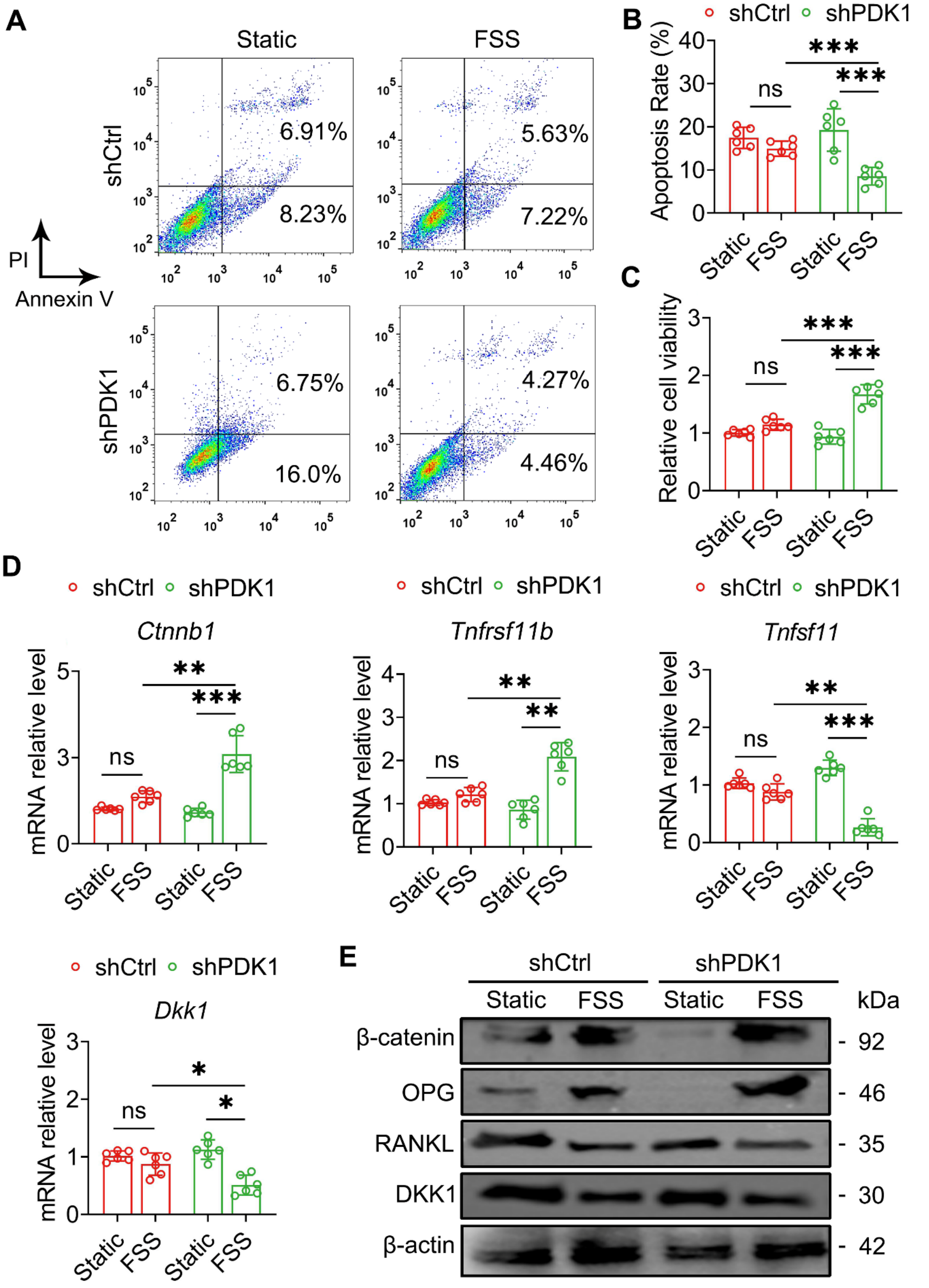

Figure S17, related to Figure 8. Effects of PDK1 silencing on cellular viability and function in response to fluid flow stimulation in MLO-Y4 osteocytic cells exposed to SMG. **(A~C)**

The Annexin V-FITC/PI apoptosis assays and CCK-8-based cell viability assays. *n*=6/group. **(D)** qRT-PCR for the gene expression of *Ctnnb1*, *Tnfrsf11b*, *Tnfsf11*, and *Dkk1*. *n*=6/group. **(E)** Western blotting analyses of the protein expression of β-catenin, OPG, RANKL, and DKK1. Graphs represent mean ± SD. **P*<0.05, ***P*<0.01, and ****P*<0.001 by 2-way ANOVA with Bonferroni's post test.

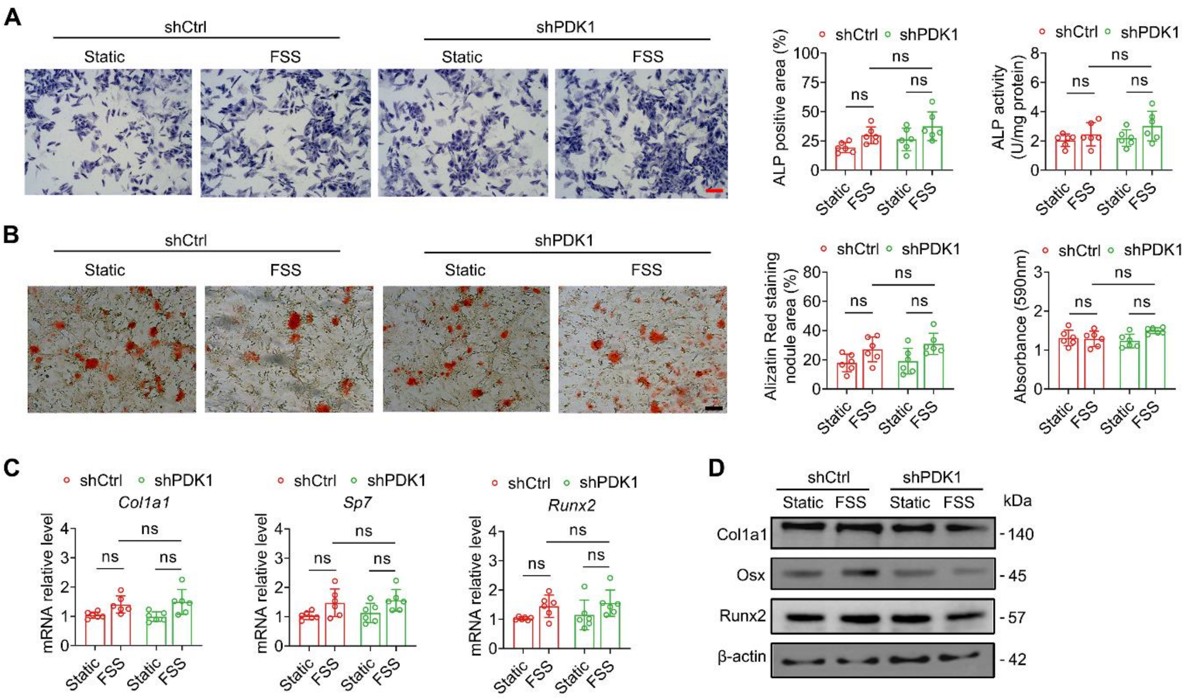

Figure S18, related to Figure 8. Effects of PDK1 silencing on cellular differentiation and mineralization in response to fluid flow stimulation in primary osteoblasts exposed to SMG. **(A)** ALP staining and ALP activity assays following osteogenic medium incubation. *n*=6/group. **(B)** Alizarin red staining following osteogenic medium incubation. *n*=6/group. **(C)** qRT-PCR analyses of the gene expression of *Col1a1*, *Sp7*, and *Runx2* following osteogenic medium incubation. *n*=6/group. **(D)** Western blotting analyses of the protein expression of Col1a1, Osx, and Runx2 following osteogenic medium incubation. Graphs represent mean ± SD. All analyses were performed by 2-way ANOVA. Scale bars: **A, B** 50 μm.
